# Glacial Refugia and Future Habitat Coverage of Selected *Dactylorhiza* Representatives (Orchidaceae)

**DOI:** 10.1371/journal.pone.0143478

**Published:** 2015-11-23

**Authors:** Aleksandra M. Naczk, Marta Kolanowska

**Affiliations:** 1 Department of Molecular Evolution, University of Gdańsk, Wita Stwosza 59, PL 80–308, Gdańsk, Poland; 2 Department of Plant Taxonomy and Nature Conservation, University of Gdańsk, Wita Stwosza 59, PL 80–308, Gdańsk, Poland; National Cheng-Kung University, TAIWAN

## Abstract

The intensively discussed taxonomic complexity of the *Dactylorhiza* genus is probably correlated with its migration history during glaciations and interglacial periods. Previous studies on past processes affecting the current distribution of *Dactylorhiza* species as well as the history of the polyploid complex formation were based only on molecular data. In the present study the ecological niche modeling (ENM) technique was applied in order to describe the distribution of potential refugia for the selected *Dactylorhiza* representatives during the Last Glacial Maximum. Additionally, future changes in their potential habitat coverage were measured with regard to three various climatic change scenarios. The maximum entropy method was used to create models of suitable niche distribution. A database of *Dactylorhiza* localities was prepared on the grounds of information collected from literature and data gathered during field works. Our research indicated that the habitats of majority of the studied taxa will decrease by 2080, except for *D*. *incarnata* var. *incarnata*, for which suitable habitats will increase almost two-fold in the global scale. Moreover, the potential habitats of some taxa are located outside their currently known geographical ranges, e.g. the Aleutian Islands, the western slopes of the Rocky Mountains, Newfoundland, southern Greenland and Iceland. ENM analysis did not confirm that the Balkans, central Europe or central Russia served as the most important refugia for individual representatives of the *Dactylorhiza incarnata/maculata* complex. Our study rather indicated that the Black Sea coast, southern Apennines and Corsica were the main areas characterized by habitats suitable for most of the taxa.

## Introduction

The Last Glacial Maximum (LGM) refers to the period between 26,500 and 20,000 years ago [1] that greatly affected the distributions and population sizes of many temperate plant species. Migration routes and the history of colonization after the LGM have been studied for various taxa, e.g. *Viola rupestris* [2], *Lathyrus vernus* [3], *Silene dioica* [4], *Calluna vulgaris* [5], and *Betula pendula* [6]. For a long time, it has been commonly assumed that during the LGM a lot of temperate plant species survived within refuge areas in the Balkan, Apennine and Iberian Peninsulas and in the Caspian and Caucasian regions (“the southern refugia hypothesis” [7–8]. It has also been established that the general view of high genetic diversity and haplotype richness in refugial areas in the south is the result of refugial persistence and accumulation of genetic variation during ice ages, in comparison with low diversity in glaciated areas in the north. Populations in previously glaciated areas are genetically depleted as a consequence of rapid postglacial colonization and the repeated bottleneck effect during stepwise migration [9–10]. The hypothesis is just a general concept and the individualistic nature of species' responses to climate change implies that the location of refugia varies according to the climatic conditions preferences as well as to the way individual species or populations adapt [11]. The incoming evidence suggests that the southern refugia for the temperate species were complemented by more northern refugia during the LGM. "The northern refugia hypothesis" assumes more complex patterns for the distribution of genetic diversity, where suitable niches were also distributed much more widely in Europe during the LGM, not only across Southern Europe, but also in Central Europe close to the line of the ice sheet [12–13]. This proposal has also been indicated in phylogeographic studies of selected *Dactylorhiza* species [14].

*Dactylorhiza* Neck. *ex* Nevski is a temperate orchid genus which includes taxa of various ploidy levels [15–18]. They are either diploids (2n = 40) or tetraploids (2n = 80). Most currently recognized *Dactylorhiza* species belong to the *Dactylorhiza incarnata/maculata* polyploid complex. The most problematic taxa within this complex belong to *D*. *majalis* s.l., which evolved by multiple and independent hybridization events between two broadly defined parental lineages: *D*. *incarnata* s.l.—recognized as the paternal lineage and *D*. *maculata* s.l.—considered to be the maternal lineage [18–29]. The taxonomic complexity of this genus is probably due to its migration history during glaciations and interglacial periods, as well as polyploidization episodes, which took place several times e.g. [18,24,28–30].

As assumed by Hedrén et al. [31], this complex must have originated before the Weichselian glaciation and its representatives are now distributed across Europe and Asia Minor [32–33]. Within this range, the allotetraploids often occupy limited occupancy areas [17,34] and many of them are restricted to those regions in more northern or western Europe that were completely covered by the ice sheet during the Weichselian glaciation. It has been postulated that numerous allotetraploid species evolved after the ice age on several, independent occasions by repeated local polyploidization events in areas where they are currently found (e.g. [24,31]). This hypothesis has been supported by molecular data, including allozyme variation [18–19,20,35] and AFLPs [25]. However, analysis of plastid DNA [26,28,36] has disclosed that some variants within the allotetraploids have not been encountered in the extant parental lineages, indicating that the allotetraploid complex may also include older taxa which currently remain unknown.

The *Dactylorhiza incarnata/maculata* polyploid complex constitutes an extremely dynamic model of polyploid speciation and extinction, in which polyploid species evolve continuously from the same set of broadly defined parental lineages. The pattern of colonization inferred for the complex representatives seems to be unusual compared with most other temperate taxa, where polyploids have proven to be strong colonizers of Arctic regions [37], whereas their diploid progenitors have remained much further south [28].

As is the case with other orchids, *Dactylorhiza* is not present in fossil material. Previous studies on past processes affecting the present distribution of the genus representatives as well as the history of the formation of polyploid complex have been based only on molecular data.

Environmental niche models, which are generated by combining species occurrence records (and/or absence data) with environmental GIS data layers have become increasingly important tools to address various issues in biogeography, ecology, evolution and conservation biology research [38–39]. Many methods have been used for modeling of species distribution (eg. BIOCLIM [40–41], DOMAIN [42], GLM [43], MaxEnt [44]). MaxEnt is considered as the most reliable machine learning programme in computing presence-only data (e.g. [45–48]). This application is particularly useful in the course of determining locations of glacial refugia of plants and animals, especially when the fossil material is poor (e.g. [49–51]. Nowadays, when the rate of climate change accelerates, it is increasingly important to understand the consequences [52] and here species distribution models based on current ecological niche constraints are used to project future species distributions (e.g. [53–55]).

While the frequent niche shifts in the polyploid complexes could be expected, the evidences for alternative patterns were reported. Findings published in the recent studies suggest that the niche conservatism may be more common between different cytotypes than previously recognized. The tendency for the niche of a taxon to be little changed over time was confirmed for several polyploid species complexes, i.e. *Claytonia perfoliata* (Portulacaceae) [56], *Larrea tridentata* (Zygophyllaceae) [57], *Houstonia* (Rubiaceae) [58] and *Heuchera cylindrica* (Saxifragaceae) [59]. Based on the frequent spatial segregation of the diploid and the polyploid cytotypes, and considering the fact that the polyploidization may drive the ecological divergence, species distribution modeling seems to be adequate approach for studies on biogeography of polyploid complexes.

Because no study revealed any ecological shifts within *Dactylorhiza* species so far, we assume that their niches have remained unchanged since the LGM and they will not transform in the predictable future. The quantified niche can be therefore projected across a geographic area for the purposes of mapping applicable climatic conditions for studied taxa and predicting its potential distribution [60]. In our research, the ecological niche modeling (ENM) technique has been applied in order to estimate distribution of suitable niches for three interesting *Dactylorhiza* species groups (*D*. *incarnata*, *D*. *maculata* and *D*. *majalis*) during the LGM and in the predictable future. Noteworthy, the studied tetraploid taxa represent fixed hybrids between known parental lineages. They should be therefore considered as separated entities with their own evolutionary history and characterized by specific habitat requirements. The aim of the study was to confront the phylogeographic insights into distribution of glacial refugia with the outcomes of the climate envelope models as well as to evaluate the future changes in the potential habitat coverage of the studied orchids.

## Material and Methods

### List of localities

**Fig 1:**
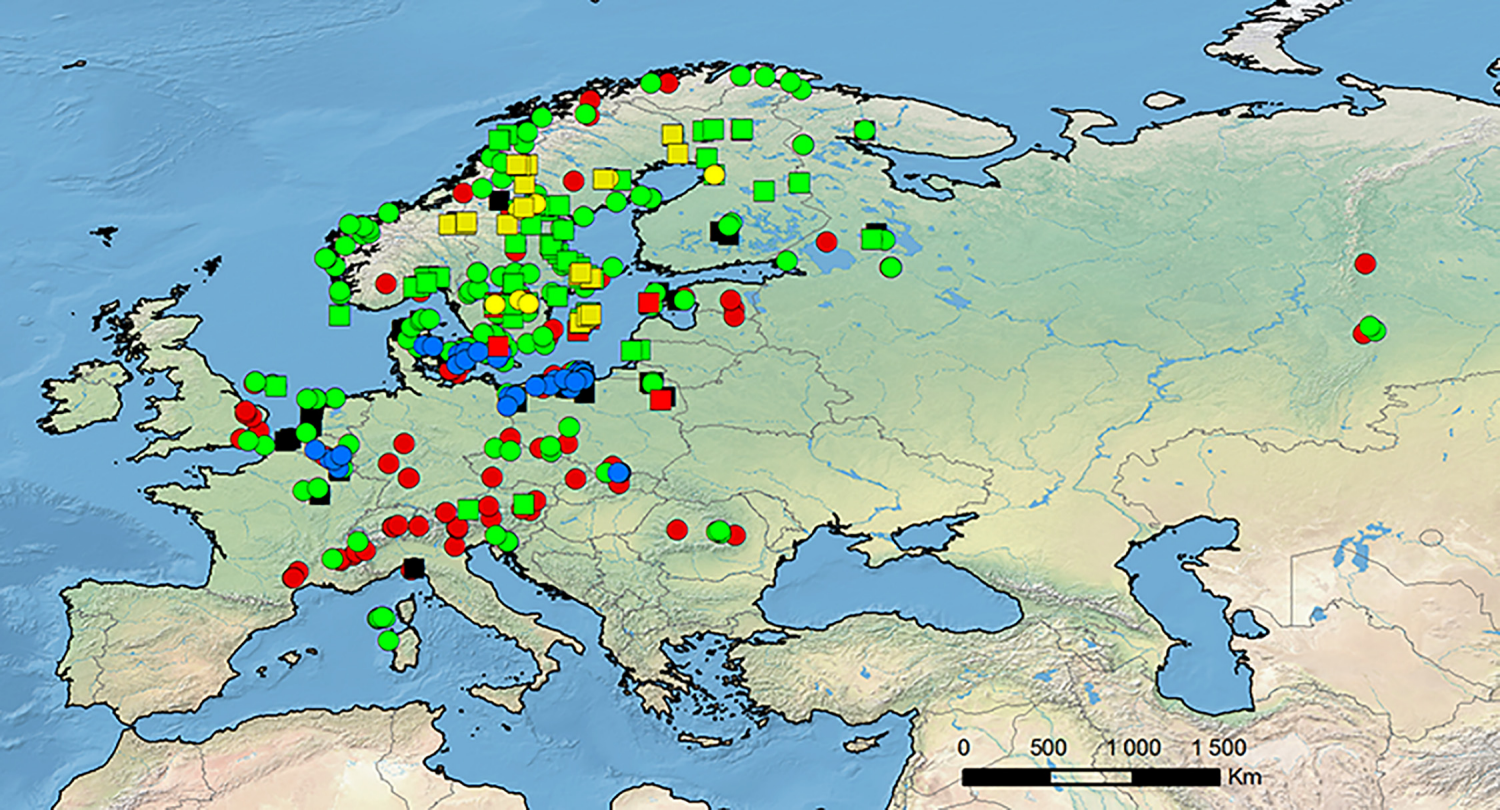
Localities of *D*. *incarnata* var. *cruenta* (yellow circle), *D*. *incarnata* var. *incarnata* (black square), *D*. *incarnata* var. *ochroleuca* (red square), *D*. *maculata* ssp. *fuchsii* (red circle), *D*. *maculata* ssp. *maculata* (green circle), *D*. *majalis* ssp. *lapponica* (yellow square), *D*. *majalis* ssp. *majalis* (blue circle), and *D*. *majalis* ssp. *traunsteineri* (green square) used in the modeling.

The potential niche modeling was performed using the maximum entropy method implemented in MaxEnt version 3.3.2 [44,61–62] and based on species presence-only observations. The list of locations in which the *Dactylorhiza* populations were found was prepared based on available literature data [14,26,29,30,35,63–68] and information gathered during the field works [69]. Only these localities which could be precisely placed on the map (Fig 1) were used in the analysis and the duplicate presence records were removed. The complete database is provided in S1 Annex. Taxonomic classification within the *Dactylorhiza incarnata/maculata* complex followed Hedrén’s concept [23].

### Ecological niche modeling

The maximum entropy method, as implemented in Maxent version 3.3.2 software, was used to create models of the suitable niche distribution [44,62,70]. Nineteen climatic variables in 2.5 arc minutes (± 21.62 km^2^ at the equator) developed by Hijmans & al. [71] as well as the altitudinal data were used as input data (S1 Table). The maximum iterations were set to 10,000 and the convergence threshold to 0.00001, thereby forcing the program not to finish before the threshold was reached. For each run, 20% of the data were used to be set aside as test points [72]. The "random seed" option was applied and it provided a random test partition and background subset for each run. The run was performed as a bootstrap with 100 replicates and the output was set to logistic. All operations on GIS data were carried out on ArcGis v. 9.3 (ESRI). Because only current location data for studied taxa were available the records were the base for all models. The contemporary species-climate relationships were projected for the LGM and three future layers using the settings described above.

The bioclimatic data for the LGM were developed and mapped by Paleoclimate Modelling Intercomparison Project Phase II [73] (PMIP2, CCSM) and downloaded from www.worldclim.org. In the projections the climatic data are extrapolated onto a modern world map, so it is easier to compare the distribution of glacial refugia with the current range of the studied taxa. For the purposes of showing the actual location of the refugial areas, we also ran the ENM using CCSM4 bioclims maps for the LGM developed by Coupled Model Intercomparison Project Phase 5 (CMIP5 [74]).

The future climatic projections related to a hypothetical climate change in 2080 with A1b (CCCMA-CGCM3 simulation), A2a (CCCMA-CGCM2) and B2a (CCCMA-CGCM2) scenarios obtained from the CIAS website (http://ccafs-climate.org; [75]). Those models were used in numerous recent studies focused on climate change impact on the distribution of various organisms (e.g. [76–78]). The A1b scenario is characterized by a balance across all energy sources (where balanced is defined as not relying too heavily on one particular energy source, provided that similar improvement rates apply to all energy supply and end-use technologies). The A2 storyline describes a highly heterogeneous future world with regionally oriented economies. The main driving forces involve a high rate of population growth, increased energy consumption, land-use changes and slow technological change. The B2 is a scenario with a general evolution towards environmental protection and social equity. The modeling for the future was performed with the same settings as for the present time. The global coverage area of the most suitable niches was calculated in order to evaluate general effects of the global warming on the potential distribution of *Dactylorhiza*. To precisely determine the possible negative effect of climate changes on the studied orchids, the same computation was made for the region of their known geographical range (within the longitude of 15.37–180.30 and the latitude of 29.21–86.98). Because most of the known populations of the species studies were found in the zones characterized by high habitat fitness of 0.7, the number was applied as the threshold value of the most suitable niches.

## Results

### Model evaluation

All the models created received high AUC (area under ROC curve) scores of 0.987–0.998 (S2 Table). The results are consistent with the outcomes of previous studies which indicated the reliable performance of the method for the purposes of developing ecological niche models based exclusively on presence-only data [52].

### Glacial refugia

**Fig 2:**
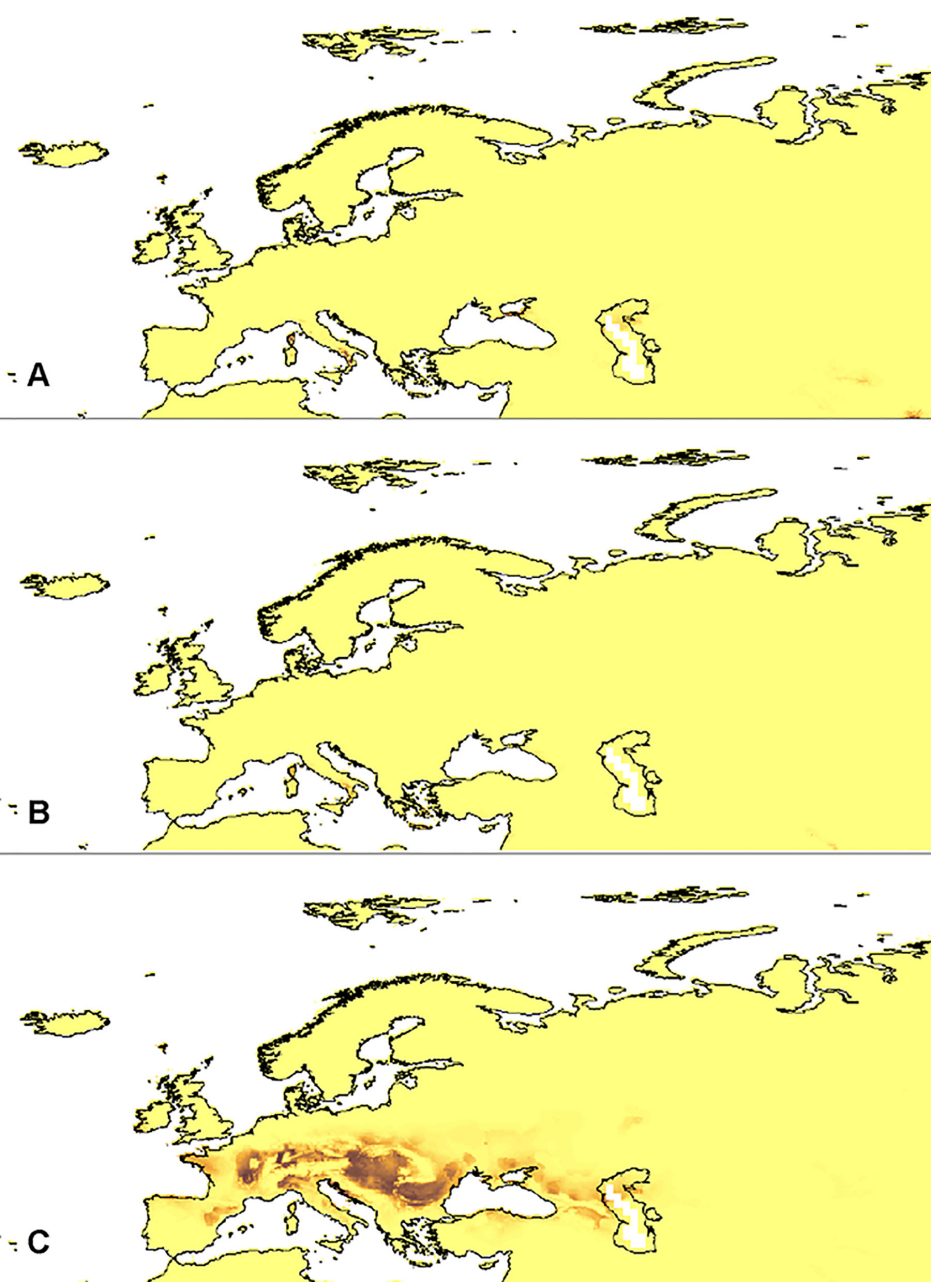
European distribution of the suitable niches of *D*. *majalis* ssp. *lapponica* (A), *D*. *majalis* ssp. *majalis* (B), and *D*. *majalis* ssp. *traunsteineri* (C) within their currently known geographical range in the LGM (PMIP2 based models).

**Fig 3:**
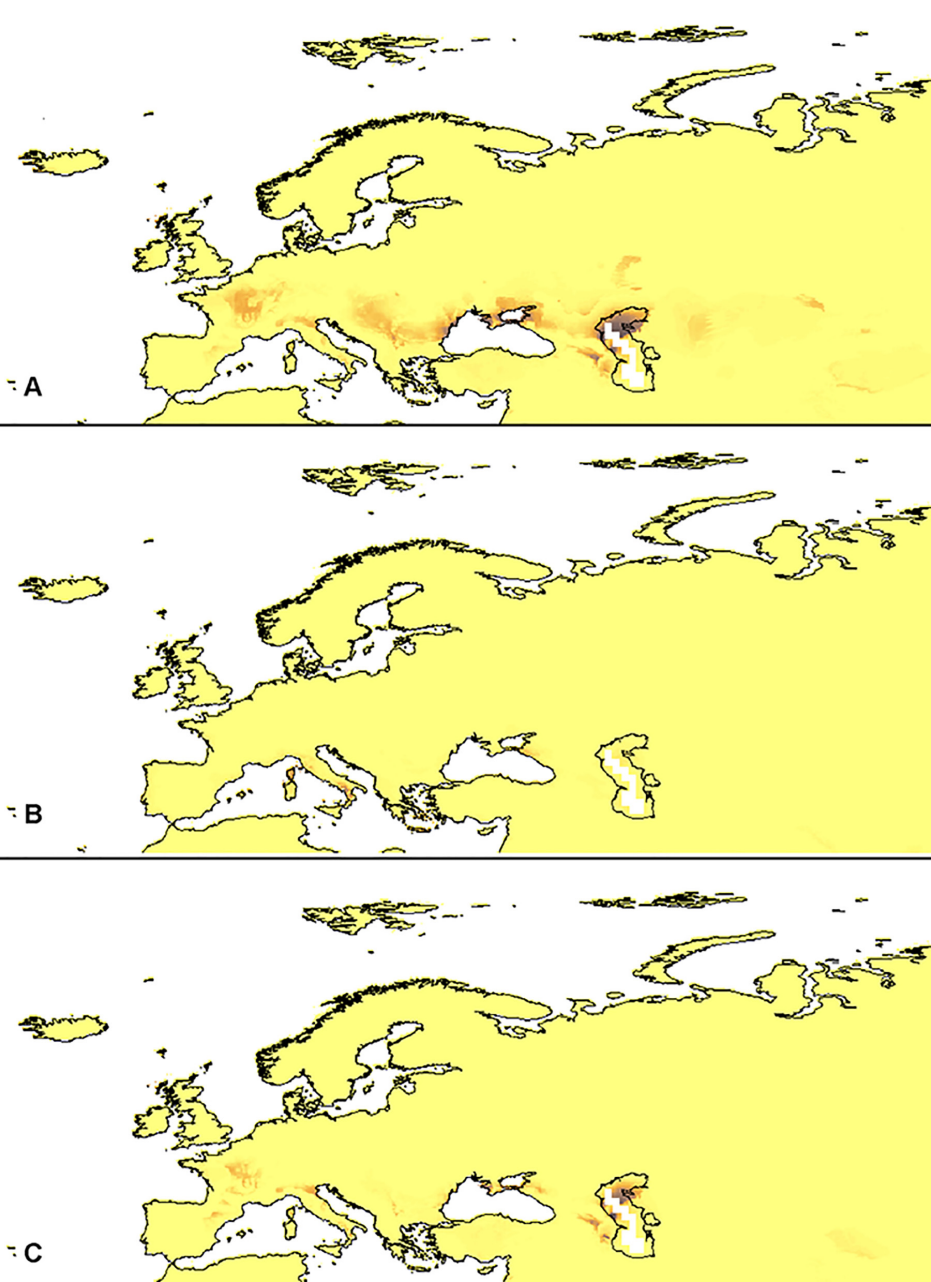
European distribution of the suitable niches of *D*. *incarnata* var. *cruenta* (A), *D*. *incarnata* var. *incarnata* (B), and *D*. *incarnata* var. *ochroleuca* (C) within their currently known geographical range in the LGM (PMIP2 based models).

**Fig 4:**
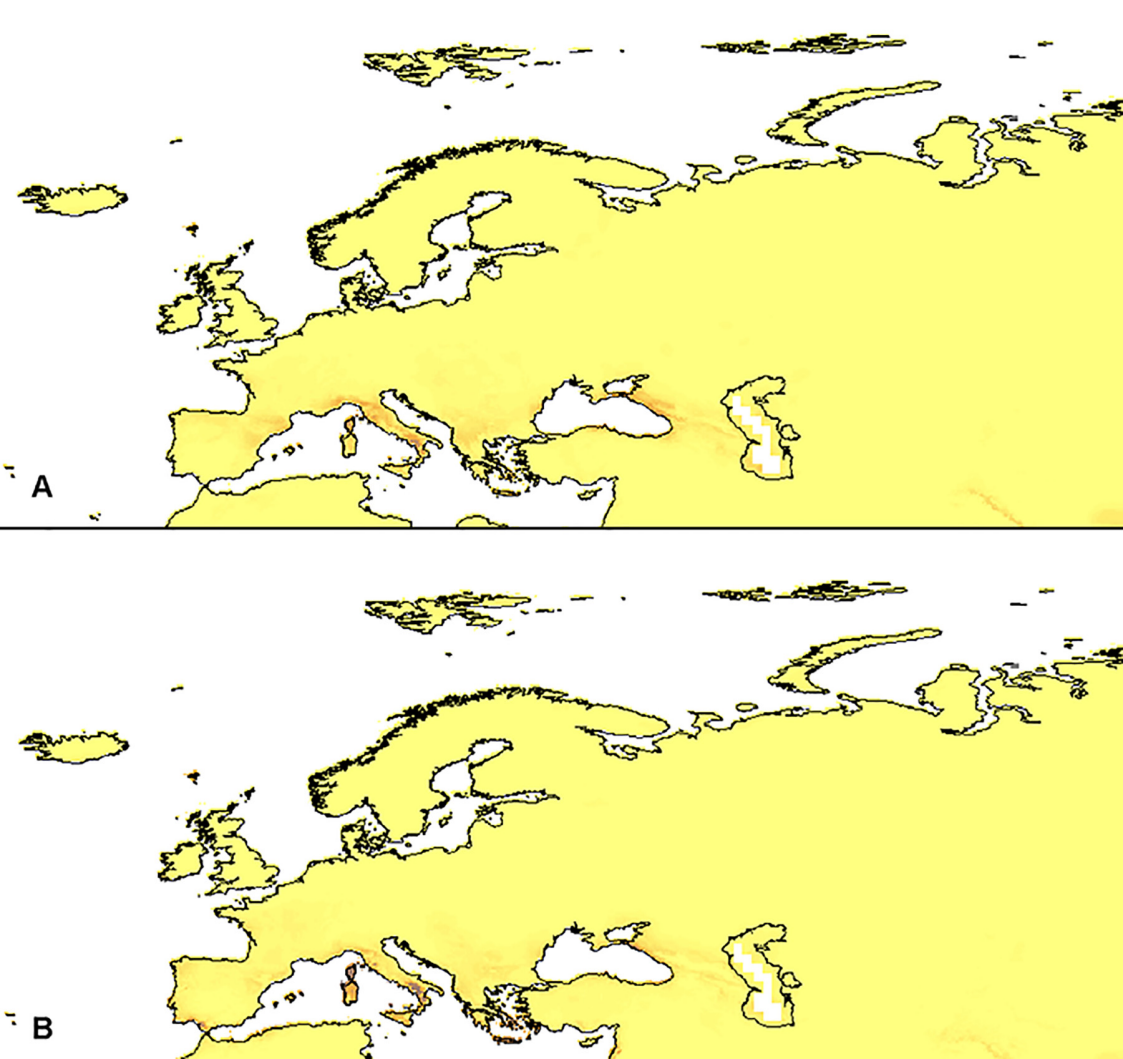
European distribution of the suitable niches of *D*. *maculata* ssp. *fuchsii* (A) and *D*. *maculata* ssp. *maculata* (B) within their currently known geographical range in the LGM (PMIP2 based models).

The models of the suitable niches of the selected *Dactylorhiza* representatives' distribution in the LGM in Europe are presented in Figs 2–4 (PMIP2 based models). The global models are presented in S1–S6 Figs (PMIP2 based models) and S4–S6 Figs (CMIP5 based models).

Within the known geographic range of the studied taxa, the most probable refugia of *D*. *incarnata* var. *cruenta* Hyl. were located on the western and northern coasts of the Caspian Sea as well as on the coast of the Sea of Azov and the western coast of the Black Sea (Danube delta). Some less suitable niches were distributed on the eastern slopes of the northern Apennines, the western slopes of the western Alps and along the Danube. The main refugia of *D*. *incarnata* var. *incarnata* were located in Corsica and the southern Apennines. Populations of *D*. *incarnata* var. *ochroleuca* (Wüstnei *ex* Boll) P.F. Hunt & Summerh could survive on the north-eastern coast of the Caspian Sea, the southern part of the Caspian Depression, the eastern slopes of the Caucasus and the southern coast of the Sea of Azov, including western Crimea. Some less suitable habitats of this taxon were located in Corsica, the eastern slopes of the northern Apennines and the western Alps.

The models created indicated the eastern part of the Black Sea, the southern Apennines and Corsica as the most probable refugia for *D*. *maculata* ssp. *fuchsii* Hyl. Additional habitats could be found in Crete and Sicily. The potential glacial range of *D*. *maculata* ssp. *maculata* included the eastern coast of the Black Sea, Crete, the Cyclades, Rhodes as well as the southern Apennines, Sicily, Corsica, Sardinia and Menorca. Some less suitable niches could be located in Andalusia.

During the LGM, populations of *D*. *majalis* ssp. *lapponica* H. Sund. could survive in Corsica, the southern Apennines, northern Sicily, and the southern coast of the Sea of Azov. Additional, fragmented refugia were located in Crete. Populations of *D*. *majalis* ssp. *majalis* in the LGM could be distributed in the southern Apennines, Corsica and Sicily. Unlike those taxa, refugia of *D*. *majalis* ssp. *traunsteineri* (Saut. *ex* Rchb. f.) H. Sund. were probably distributed on the Pannonian Plain, Dinaric Alps as well as along the Danube and at the lower altitudes along the range of the Alps. Numerous less suitable niches were also located in the eastern Pyrenees and the slopes of the Caucasus.

When considering the global distribution of the potential glacial refugia of the studied taxa, several areas were indicated by ENM analysis to be well outside their current geographical ranges (Figs 2–4). The most important regions that could be characterized by climatic conditions suitable for the studied *Dactylorhiza* representatives are the Aleutian Islands and the Alaska Peninsula in North America, Patagonia in South America and the Kamchatka shore.

Additionally, the south-eastern slopes of the Himalayas were indicated by MaxEnt as suitable for *D*. *majalis* ssp. *lapponica*, *D*. *majalis* ssp. *majalis* and *D*. *incarnata* var. *incarnata*. In the LGM period, the Zambezi River Basin could have served as a refugium for *D*. *majalis* ssp. *majalis*, *D*. *maculata* ssp. *maculata* and *D*. *incarnata* var. *incarnata*. Suitable niches of *D*. *majalis* ssp. *traunsteineri* were also located on the north shore of the Gulf of Mexico and the east of the southern Appalachian Mountains. In the glacial period, suitable habitats of *D*. *majalis* ssp. *traunsteineri*, *D*. *incarnata* var. *cruenta* and both representatives of *D*. *maculata* complex were also located in southern Japan.

### Current potential distribution

**Fig 5:**
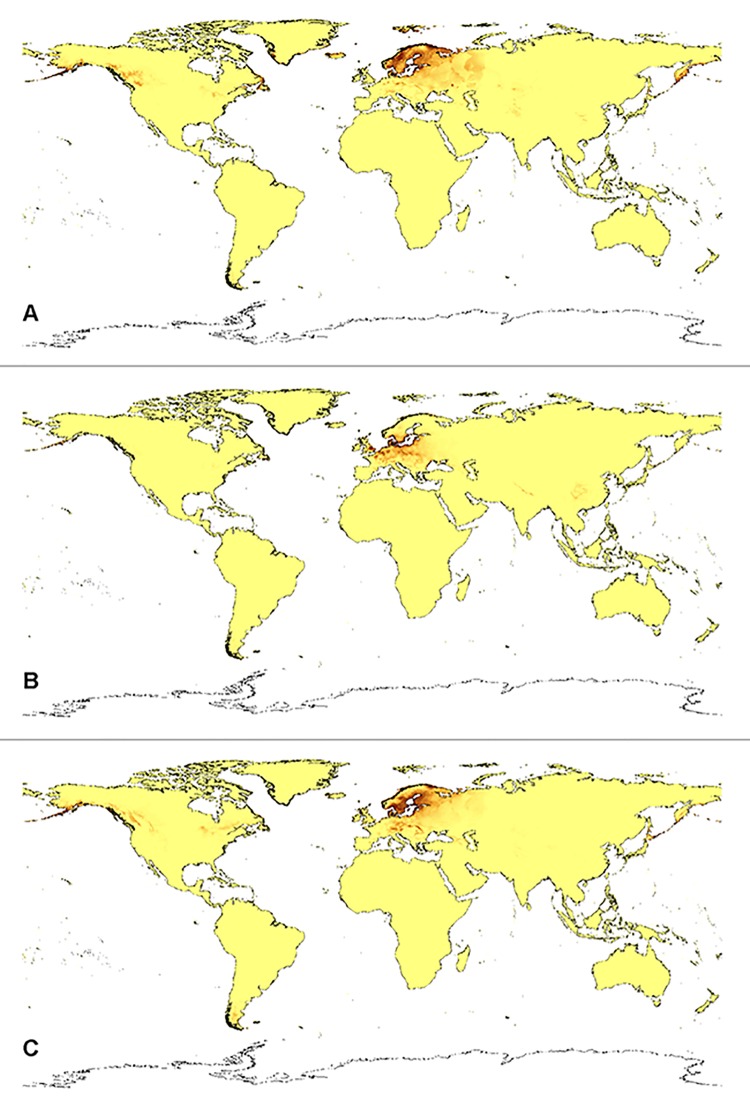
Current distribution of suitable niches of *D*. *majalis* ssp. *lapponica* (A), *D*. *majalis* ssp. *majalis* (B), and *D*. *majalis* ssp. *traunsteineri* (C).

The distribution of the suitable niches for allotetraploid representatives of *D*. *majalis* in Europe and Asia Minor indicated by ENM analysis corresponds to the known geographical range of those taxa [17,79]; however, several regions where none of these orchids have been found so far, have also been specified as potentially suitable. For *D*. *majalis* ssp. *lapponica*, the suitable niches outside its geographical range are located in the southern parts of Svalbard, the Kamchatka Peninsula, the Aleutian Islands, the Alaska Peninsula, the eastern part of Newfoundland and south-western Greenland. Some less suitable habitats are also found in Iceland (Fig 5A). Some habitats of lower suitability potentially suitable for *D*. *majalis* ssp. *majalis* were indicated in the models in the Aleutian Islands, the Alaska Peninsula and small areas in eastern Newfoundland (Fig 5B). The Kuril Islands, Kamchatka Peninsula, Aleutian Islands, Alaska Peninsula, northern Coast Mountain and northern Rocky Mountains were indicated as potentially suitable for *D*. *majalis* ssp. *traunsteineri*. It is noteworthy that the models did not indicate numerous areas in central Asia as suitable for this taxon, despite known occurrence reports of *D*. *majalis* ssp. *traunsteineri* in these regions (Fig 5C).

**Fig 6:**
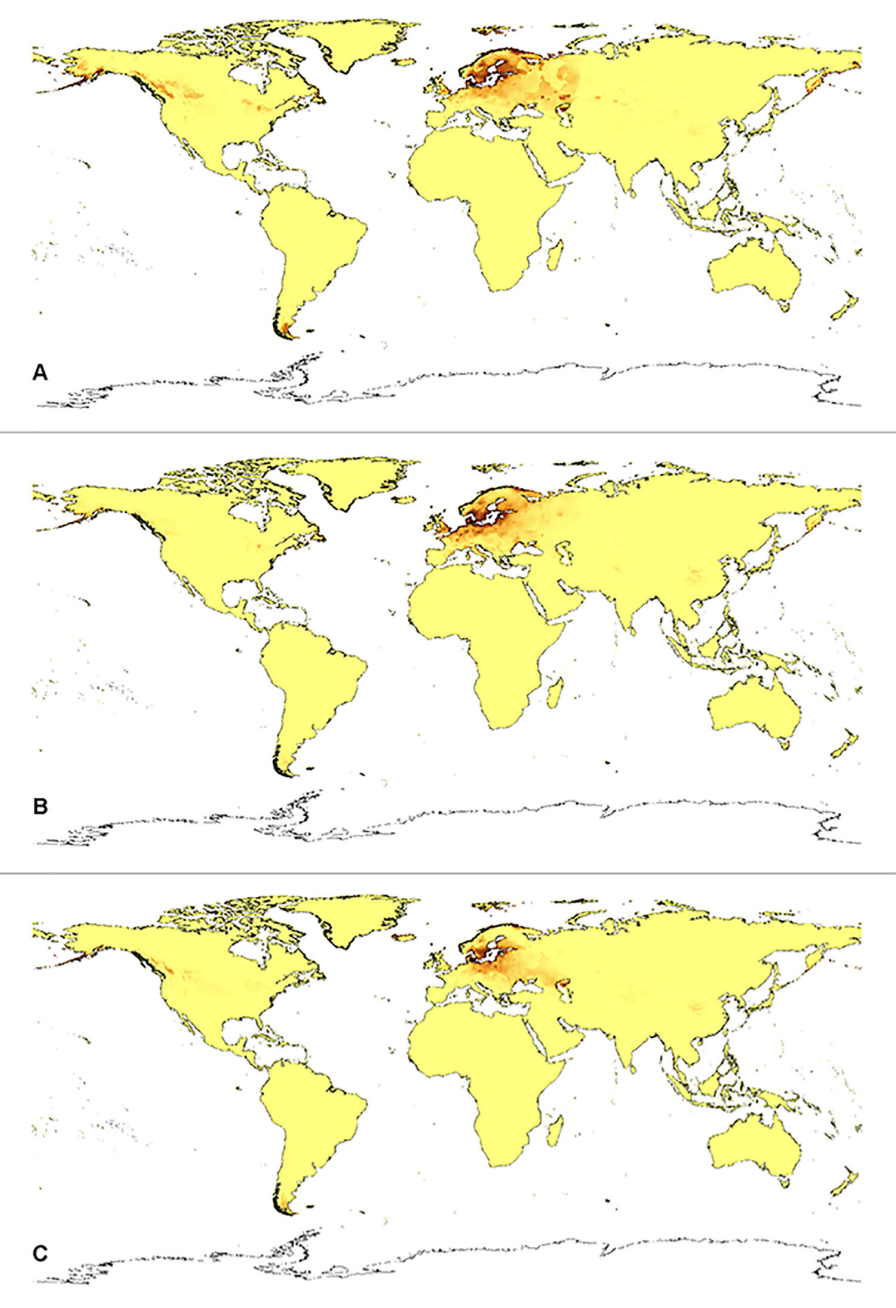
Current distribution of suitable niches of *D*. *incarnata* var. *cruenta* (A), *D*. *incarnata* var. *incarnata* (B), and *D*. *incarnata* var. *ochroleuca* (C).

In all the three models created for the diploid representatives of *D*. *incarnata*, the Aleutian Islands and Alaska Peninsula were indicated as potentially suitable for those taxa; however, none of these have been found in the regions (Fig 6). Additionally, habitats suitable for *D*. *incarnata* var. *cruenta* are also located along the shores of the Black and Caspian Seas and the eastern margins of the Kamchatka Peninsula. Some less appropriate habitats are also located in the Rocky Mountains and Patagonia (Fig 6A). Among the areas indicated by the ENM analysis as suitable for *D*. *incarnata* var. *ochroleuca*, it was only the northern Caspian region that this taxon was not observed in. Additional habitats were indicated in the Aleutian Islands and the Alaska Peninsula (Fig 6C). Less suitable niches are located in the northern Rocky Mountains and northern Iceland.

**Fig 7:**
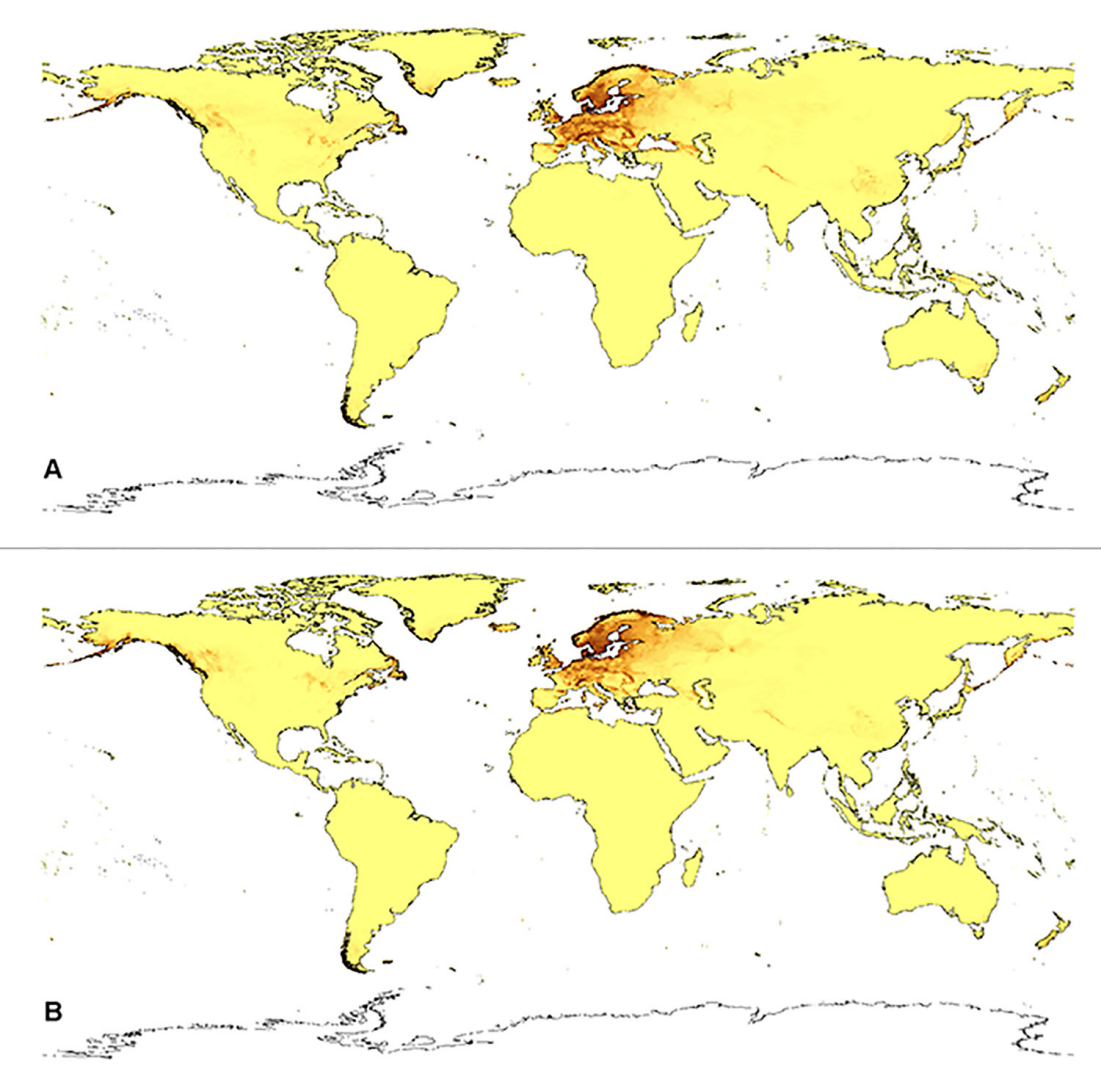
Current distribution of suitable niches of *D*. *maculata* ssp. *fuchsii* (A) and *D*. *maculata* ssp. *maculata* (B).

Models created for the *D*. *maculata*-complex indicated several regions suitable for the studied taxa. The Black Sea, south-western Himalayas, Kuril Islands, Aleutian Islands and Alaska Peninsula are potentially suitable for *D*. *maculata* ssp. *fuchsii* (Fig 7A) and the Kuril Islands, Kamchatka Peninsula, Aleutian Islands, Alaska Peninsula, and the eastern part of Newfoundland are suitable for *D*. *maculata* ssp. *maculata* (Fig 7B). Noteworthy is the fact that the models did not indicate numerous Asian areas as suitable for the latter taxon, the occurrence of which had been reported in previous publications (e.g. [17]).

### Future habitat coverage

The models of the potential distribution of *Dactylorhiza* representatives in 2080 within known geographical range are presented in S7–S9 Figs.

Firstly, the evaluation of the global coverage of their suitable habitats was calculated (Table 1). The habitats of almost all taxa will decrease in scenario A1b. In scenario A2a a loss of habitats will be observed for *D*. *incarnata* var. *ochroleuca*, *D*. *maculata* ssp. *fuchsii*, *D*. *maculata* ssp. *maculata* and *D*. *majalis* ssp. *majalis* and negative changes will affect *D*. *maculata* ssp. *maculata* and *D*. *majalis* ssp. *majalis* in scenario B2a. The most interesting change may be observed with regard to the coverage area of suitable niches for *D*. *incarnata* var. *incaranta* in A2a and B2a scenarios. According to the models, the area of suitable habitats of this orchid will increase almost two-fold. However, there are no substantial changes concerning the areas of occurrence of *D*. *incarnata* var. *cruenta*, *D*. *majalis* ssp. *lapponica* and *D*. *majalis* ssp. *traunsteineri* in any scenario.

**Table 1:** The global coverage [km^2^] of the most suitable habitats for *Dactylorhiza* representatives (suitability of over 0.7) in the present time and in 2080 (A1b, A2a, B2a).

Taxon	A1b^a^	A2a^b^	B2a^c^	Present time
*D*. *incarnata* var. *cruenta*	9740155.92	9920596.44	9844602.14	9824906.32
*D*. *incarnata* var. *incarnata*	10163907.92	18911014.00	18412608.14	9926001.44
*D*. *incarnata* var. *ochroleuca*	9734123.94	9863541.26	9907040.70	9874243.16
*D*. *maculata* ssp. *fuchsii*	9691121.76	9308880.16	9925698.76	9888988.00
*D*. *maculata* ssp. *maculata*	9658237.74	9666583.06	9349244.70	10468512.10
*D*. *majalis* ssp. *lapponica*	9913721.28	9922326.04	9971316.96	9897398.18
*D*. *majalis* ssp. *majalis*	9729389.16	9963144.60	9810399.30	10028242.42
*D*. *majalis* ssp. *traunsteineri*	9988894.02	9989931.78	10116776.32	9970127.86

^a^ CCCMA-CGCM3 climate change simulation for 2080

^b^ CCCMA-CGCM2 climate change simulation for 2080

^c^ CCCMA-CGCM2 climate change simulation for 2080

It is noteworthy that the suitable habitats of some taxa are located outside their known geographical ranges, e.g. the Aleutian Islands (*D*. *incarnata* var. *cruenta*, *D*. *maculata* ssp. *maculata*), the western slopes of the Rocky Mountains (*D*. *incarnata* var. *cruenta*, *D*. *maculata* ssp. *maculata*), Newfoundland (*D*. *maculata* ssp. *maculata*, *D*. *majalis* ssp. *lapponica*), southern Greenland and Iceland (*D*. *majalis* ssp. *lapponica*), Tierra del Fuego (*D*. *incarnata* var. *cruenta*, *D*. *incarnata* var. *ochroleuca*), and New Zealand (*D*. *maculata* ssp. *fuchsii*, *D*. *maculata* ssp. *maculata*).

To determine the precise possible negative effect of climate changes within the known geographical range of each taxon, the coverage of the most suitable niches was calculated for Eurasia (Table 2). Within the region in A1b scenario only the coverage of *D*. *incarnata* var. *incarnata* habitats will slightly increase (by about 2%). The available niches of all other taxa will decrease. The changes predicted in the scenario will be the most destructive for niches of the studied orchids. In A2a scenario a reduction of suitable habitats will not be observed only in *D*. *maculata* ssp. *fuchsii* (0.4% increase). In B2a scenario the coverage of suitable niches of *D*. *maculata* ssp. *fuchsii* and *D*. *maculata* ssp. *maculata* will not be negatively affected.

**Table 2:** The coverage [km^2^] of the most suitable habitats for *Dactylorhiza* representatives (suitability of over 0.7) at present time and in 2080 (A1b, A2a, B2a) in Europe and Asia.

Taxon	A1b^a^	A2a^b^	B2a^c^	Present time
*D*. *incarnata* var. *cruenta*	821149.22	1087658.96	1051358.98	1120521.36
*D*. *incarnata* var. *incarnata*	664512.32	599933.38	625596.32	651670.04
*D*. *incarnata* var. *ochroleuca*	316365.46	363864.60	379344.52	421417.04
*D*. *maculata* ssp. *fuchsii*	1246349.76	1287341.28	1450550.66	1282411.92
*D*. *maculata* ssp. *maculata*	1011621.42	1095334.06	1247171.32	1100998.50
*D*. *majalis* ssp. *lapponica*	897100.28	885101.18	787097.72	997806.24
*D*. *majalis* ssp. *majalis*	138022.08	187423.78	179294.66	222707.62
*D*. *majalis* ssp. *traunsteineri*	772633.94	858486.96	861513.76	918352.74

^a^ CCCMA-CGCM3 climate change simulation for 2080

^b^ CCCMA-CGCM2 climate change simulation for 2080

^c^ CCCMA-CGCM2 climate change simulation for 2080

## Discussion

### Potential glacial refugia within the known geographical range

The almost complete distinction between diploid lineages of *D*. *incarnata* s.l. and *D*. *maculata* s.l. at allozyme loci suggests that the lineages characterized with a long period of separated history and that gene flow between them did not exist for a long time [18,23]. However, according to our study, suitable niches of the taxa overlapped during the LGM in Europe.

In contrary to the genetic research results [14,31] the ENM analysis did not confirm that the Balkans, central Europe or parts of central Russia located between the Fennoscandian ice sheet and the Urals served as the most important refugia for most representatives of the *Dactylorhiza incarnata/maculata* complex during the LGM. Also our analysis did not point out any areas of sheltered topography in central Europe to be refugial for *D*. *maculata* ssp. *fuchsii* which was postulated by Ståhlberg and Hedrén [14]. Our study indicated the Black Sea coast, southern Apennines and Corsica as the main areas characterized by habitat suitable for all taxa, except *D*. *majalis* ssp. *traunsteineri*.

Below, we present a more detailed discussion of the differences and similarities between the results obtained in the course of our study and data collected by other authors, mainly based on the distribution of plastid DNA haplotypes.

#### *D*. *majalis* complex (ssp. *lapponica*, ssp. *majalis*, ssp. *traunsteineri*)

Numerous allotetraploids have originated by hybridization between parental lineages related to present-day *D*. *incarnata* s.l. and *D*. *maculata* s.l. Within the *D*. *majalis* core complex (e.g. *D*. *majalis* ssp. *majalis*, *D*. *majalis* ssp. *lapponica*, and *D*. *majalis* ssp. *traunsteineri sensu* Nordström and Hedrén [80]) presence of markers which are no longer found in the parental lineages was notified [28,36]. This suggests that some allotetraploids may be relatively old and those most probably originated long before the last glaciation. It is generally accepted that allotetraploids in *D*. *majalis* s.l. are often narrow endemics, where populations were established by a single founder event or immigrated by long-distance dispersal. Subsequently, the initial population of *Dactylorhiza* expanded to form the present metapopulation, where genetic drift and natural selection driven by ecological conditions caused the evolution of distinct subspecies within the group [35]. The analysis of plastid DNA, the results of which were presented in previous studies [30–31,80–81], indicated several migration routes for the allotetraploid representatives of *Dactylorhiza*.

Given the distribution of the H1 haplotype, Scandinavian populations of *D*. *majalis* ssp. *traunsteineri/lapponica* originated in continental Europe and entered the Swedish and Norwegian mainland probably *via* Denmark, while the analysis of the H4 haplotype indicated that other routes of migration might have occurred *via* Estonia and Finland to northern Sweden and Norway [30,80–81]. The high frequencies of the two haplotypes may be explained by the rapid expansion of populations carrying the haplotypes after the last glaciations.

Our study confirmed the location of glacial refugia of *D*. *majalis* ssp. *traunsteineri* around the Alps and the area also maintained most of the recognized plastid haplotypes and the largest number of unique haplotypes for this taxa [82]. However, the suitable niches of other *Dactylorhiza* representatives were located further to the south in the ENM analysis (e.g. Corsica, the southern Apennines, the southern coast of the Sea of Azov), but we do not have molecular data for the remaining subspecies of *D*. *majalis* studied, in order to be able to compare them. Several of the plastid haplotypes that were found in Greece have not been detected in material outside this country. Other findings suggest that also northern parts of the Balkans are characterized by high overall diversity [31]. Hereby parts of the Balkans could also served as an important refugium from which northern European allotetraploids were recruited. *Dactylorhiza* populations could have also survived in other areas and it is suggested that allotetraploid populations in eastern Europe and Russia should be collected for extended studies [31].

#### *D*. *incarnata* complex (var. *cruenta*, var. *incarnata*, var. *ochroleuca*)

Allozyme data clearly indicated that Turkish populations of *D*. *incarnata* s.l. were far more genetically variable than populations from northern Europe, because the species appears to have lost genetic variation during its recolonization from southern refugia after the Weichselian glaciation [18,22–23,64,83]. Pillon et al. [28] revealed a higher level of genetic diversity in the Caucasus and the Mediterranean Basin than in western Europe using plastid and nuclear DNA sequence data. Numerous studies have presented a similar picture of differentiation, based on the plastid polymerase chain reaction-restriction fragment length polymorphisms (PCR-RFLPs; [24,26]), nrDNA internal transcribed spacer/external transcribed spacer (ITS/ETS) sequence data [27,84–85] and length-variable ITS fragments [28,86]. Generally, the common occurrence of several haplotypes in southern Scandinavia and also in adjacent areas to the south and the east of the Baltic Sea suggests that *D*. *incarnata* s.l. has been dispersed across the Baltic on repeated occasions. Furthermore, all Scandinavian haplotypes were present in the material analyzed from Turkey further to the southeast. The pattern obtained suggests that the principal direction of migration into Scandinavia was from the south or the southeast, although it is possible that additional migrants may have come from other directions [84].

Our study confirmed previously published hypothesis on the location of glacial refugia of *D*. *incarnata* s.l. [22,28,84,87]. The suitable niches of *D*. *incarnata* s.l. during the LGM were located in Corsica, the southern Apennines, the southern Balkans and the northeastern coast of the Black Sea. In addition to the available literature data [84] the location of habitats preferred by *D*. *incarnata* var. *cruenta* was indicated in our test around the Alps.

#### *D*. *maculata* complex (ssp. *fuchsii* and ssp. *maculata*)

A large number of regionally focused studies based on morphological and cytological data [29,88–94], as well as on plastid DNA and nuclear ribosomal DNA markers [24,26–28,36,86,95], have indicated several potential sources of tetraploid populations of *D*. *maculata* s.l. According to the results, central Europe could serve as the source of the southern/western lineage of *D*. *maculata* ssp. *maculata*, as well as for *D*. *maculata* ssp. *fuchsii*. This genealogical lineage is currently distributed from Portugal in the south, through the British Isles, western and central Continental Europe, to the Scandinavian mountain range in the north, and also from Iceland in the west to Romania in the east and is characterized by the Group II of haplotypes. However, the northern/eastern lineage of *D*. *maculata* ssp. *maculata* may have survived the LGM in central Russia and also west of the Urals and at present, it is spread from northern Norway to the Urals in the east [14]. It is also expected that the pattern is additionally complicated by the areas of sheltered topography in central Europe which may have provided suitable habitats for the more thermophilous *D*. *maculata* ssp. *fuchsii* during the LGM [13–14]. The Mediterranean region and the Caucasus have not contributed to the northward migration of either *D*. *maculata* ssp. *fuchsii* or *D*. *maculata* ssp. *maculata* [14]. However, the distribution of genetic diversity suggests that northern Europe has been colonized from the south to the north while, there is no support for a north-eastern recolonization route for *D*. *maculata* ssp. *fuchsii* [14,29]. For Scandinavia, the average gene diversity over loci gradually decreases from the south to the north, in accordance with a stepping-stone model of gene dispersal [14]. Nonetheless, our study did not indicate the presence of suitable niches in central Europe or central Russia either for diploid *D*. *maculata* ssp. *fuchsii* or for autotetraploid *D*. *maculata* ssp. *maculata*. The suitable niches obtained for *D*. *maculata* representatives were located more to the south in the ENM analysis (e.g. Corsica, the southern Apennines, the eastern coast of the Black Sea). However, Ståhlberg & Hedrén [14] received a low frequency of private haplotypes for these areas which is reflected in low values in terms of haplotype richness and gene diversity [14]. This limited diversity indicates a history of small population size and a consequent loss of variation through genetic drift [96]. It should be stressed that in the previous molecular studies [14] only two populations were sampled in southern Apennines and no data from the Corsica were included in the analysis. Extended sampling of *D*. *maculata* s.l. in broader areas of the Balkans, as well as in central Europe and central Russia would be needed to resolve the differences at a further stage.

### Glacial refugia outside known geographical range

The ENM analysis indicated several additional potential refugial areas for *Dactylorhiza* representatives, all of which were located near the ice sheet borders. The Aleutian Islands, the edges of the Alaska Peninsula and Kamchatka shore indicated in the models, correspond to the ice-free part of Beringia which served as an important refugium for numerous plants during the LGM [97]. The other region of North America where *Dactylorhiza* populations could hypothetically survive was located between the Cordilleran Ice Sheet and the Laurentide Ice Sheet [98]. The refugia in Patagonia were located both east and west of the glacier [99]. While the climatic conditions of those areas were apparently appropriate for the studied orchids, the absence of their populations near the mentioned potential refugia bring into question their actual usefulness for those plants. Considering the current geographical ranges of the studied taxa, we would argue that those regions actually contribute to the current patterns of *Dactylorhiza* diversity and distribution.

### Ecological niche modeling vs molecular analysis

The three major southern Mediterranean peninsulas (Iberian, Apennine, Balkan) have for a long time been considered as main Pleistocene refugia for European flora and fauna [100]. The recent studies indicated that the paradigm is too simplistic to explain observed diversity patterns [101–102]. Some authors have postulated that presence of refugia within refugia [103] or multiple northern refugia [13,104] should be considered as additional factors affecting current distribution of European taxa. Traditionally, paleoecological data [105–106] and analysis of endemic taxa concentration [107] were used to localize glacial refugia for various organisms.

Advances in molecular techniques allowed to reveal historical patterns of population divergence, including identification of potential refugial sites [108]. Many of these studies have been devoted to the Pleistocene refugia. The molecular analyses are useful in pointing out regions characterized by the presence of multiple lineages or high genetic diversity which are indicators of putative glacial refugial areas [9]. However, extinction of genetic variants, incomplete sampling and large-scale postgalcial range shifts can interrupt phylogenetic patterns and affect conclusions on the past distribution of studied organisms [109]. Moreover, the phylogeographic approaches have one inherent weakness: candidate refugia outside the present species range can rarely be identified [110]. This limitation of phylogeographic approach can be overcome through the use of species distribution models. Such projections assume that a species is in equilibrium with its environmental requirements—its distribution is determined mainly by the environment, not other factors such as competition or dispersal limitation. The main advantage of the ENM approach is providing linkage to bioclimatic and geographical factors affecting the distribution of glacial refugia. This technique gives first approximation of the spatial distribution and extent of potential Pleistocene refugia. The inadequacy of ENM models may be the result of insufficient samples (location records) included in the analysis or biased samples of occurrence data [111], however we believe that we can avoid these troubles by removing duplicated records from the database. It is inappropriate to ask the question which of the two approaches (ENMs or phylogeography) is more reliable in describing distribution of glacial refugia. The rigorous population genetic nature of the phylogeographic approaches is made more explicit spatially by the ENM approaches resulting in more quantitative product [112].

Obviously the niche analysis would be more reliable with incorporation of the biotic factors, such as pollinators or mycorrhizal fungi; however those components would not be useful in our study. Unlike numerous other orchids [113] *Dactylorhiza* representatives do not show high pollinator specificity [114–116]. The published data on the symbiotic relationship indicated that the roots of *Dactylorhiza* representatives contain several fungi belonging to both asco- and basidiomycetes [117–118]. It would be difficult to obtain occurrence data on all possible pollinators and/or mycorrhizal fungi to include those information into any analysis. For *Dactylorhiza* representatives the climatic factors seem to be the most reliable input data for the niche distribution modeling.

### Future habitat coverage

Our research indicated that in the next 65 years the coverage of suitable niches of most of the studied orchids will decrease and that the most damaging niche modifications will be observed in the A1b scenario of climate change. Clearly, the ENM analysis includes only climatic factors and some important ecological relationships, such as pollinator and mycorrhizal fungi occurrence, are omitted.

Orchidaceae is a group within the flora of Europe which is particularly vulnerable. It is caused, inter alia, by their specific biology, which makes them very sensitive to changes in their habitat, but also to the direct destruction of the plants, and of the localities occupied by them. At present, the most frequent reasons for the extinction of orchid populations are cessation of mowing, intensive fertilization, and fertilizer run-off from nearby fields and slopes. In addition, excessive fertilization of meadows and areas adjacent to the locality can lead to an imbalance in the mycoflora and to the extinction of pollinators [119–120]. Currently, the number of *Dactylorhiza* populations is decreasing due to habitat loss related mainly to the drainage of wetlands and the excessive fertilization of wet meadows. However, several taxa have shown the ability to colonize disturbed and anthropogenic sites, e.g. *D*. *maculata* ssp. *fuchsii* and *D*. *majalis* spp. *praetremissa* (Druce) D.M. Moore & Soó [121].

It has been hypothesized that each allotetraploid taxon occupies a narrow niche, has its own range and is a good indicator of specific habitats [18]. The higher expansion potential of allopolyploids may be explained by their recent origin. It has been suggested that diploids are characterized by lower genetic variation in the north part of their ranges due to the loss of alleles during their recolonization from southern refugia [84]. Allopolyploids are variable in their genetic aspect as they arose from species with different allele compositions. Allotetraploids may express the highly heterozygous genome of divergent parents with unique properties. This would explain their ability to grow in a broad spectrum of habitats of intermediate conditions preferred by their diploid progenitors [18,23,122]. External factors such as the preadaptation of the floral morphology to available pollinators, a suitable phenology, competition with existing allotetraploids and the ability to colonize previously unexploited habitats may determine which allotetraploid derivatives can multiply and spread [18,123].

The ENM analysis indicated, however, that the future available habitat coverage will not be related to the ploidy level of the studied taxa. An increase in the potential global range will be observed in *D*. *incarnata* var. *incarnata*, *D*. *majalis* ssp. *lapponica* and *D*. *majalis* ssp. *traunsteineri*. The most surprising results were obtained for *D*. *incarnata* var. *incaranta*, which demonstrated a two-fold increase of coverage area of suitable niches for this taxon; however, only on a global scale. In its currently known geographic area of occurrence, the increase will be not substantial. This orchid is characterized by a relatively narrow ecological amplitude and prefers a habitat with neutral or slightly alkaline pH, but these habitats like others have been—and indeed still are—subject to human pressure [124]. The available research results suggest that, as a result of intensive drainage work, a reduction in the number of individuals of *D*. *incarnata* in the locality can occur, and this can be observed a year or two after conducting the work, and if the unfavorable conditions remain, the total disappearance of the population occurs within 4–6 years [125]. A lowering of the groundwater level leads to a number of processes changing, e.g., the structure, thermals, and pH of the soil, which usually becomes acidified. We should keep in mind the fact that the groundwater level is a limiting factor for the occurrence of this taxon but such hydrological factors are not taken into account in the ENM analysis. As indicated in the analysis, the potential habitats of some *Dactylorhiza* representatives will also be distributed far outside their currently recognized geographical range; however, the chance of their appearance in the locations is unlikely. While a single representative of the genus, *D*. *aristata* (Fisch. *ex* Lindl.) Soó occurs in the Aleutian Islands, the migration of the European taxa to this region is difficult to imagine. Also, expansion to other areas indicated by the models as suitable for some of the studied taxa, e.g. southern Greenland, Tierra del Fuego and New Zealand appears to be improbable. These regions may not be inhabited by representatives of the genus, e. g. due to unfit soils, the lack of mycorrhizal fungi or preferred pollinators, and other ecological factors, which were not included in the selected model. It should be emphasized that our ENM analysis allowed us to identify only the climatic niches suitable for the studied orchids, not their realized niches, which in our opinion are restricted in distribution to Europe and temperate Asia.

A question arising in the context of potential biological invasions is: could the studied taxa be able to survive and spread in the areas indicated by the ENM analysis when introduced or accidentally transferred? Temperate orchids seems to have relatively small invasive potential in comparison to the tropical and subtropical taxa. So far, the only successful Orchidaceae representative native for Europe, north Africa and south-west Asia that has spread outside its range is *Epipactis helleborine*. In our opinion, the invasive potential of *Dactylorhiza* is poorer than in *E*. *helleborine* mainly due to its habitat requirements and pollinator limits. While *E*. *helleborine* grows in forest litter where the competition for pollinator service is relatively low, *Dactylorhiza* representatives occur in various open or semi-open vegetation areas with numerous other plants which are often in flower at the same time. In a newly inhabited region, these orchids would probably vanish due to the high level of pollinator competition with native plants [126–127].

Within the known geographical range of the studied taxa a general habitat loss will be observed as a result of the predicted climatic changes. While the ENM analysis was used in the conservation planning and assessment of possible changes in the suitable niche coverage of numerous taxa (i.a. [128–130]), it is worth noting that the process investigated in our research may be intensified by local anthropogenic pressures [131–133].

## Supporting Information

S1 Annex List of localities used in the ecological niche modeling. (DOC)

S1 Table Variables used in the ecological niche modeling. (DOC)

S2 Table The average training AUC for the replicate runs measured for Last Glacial Maximum (LGM), future climate change scenarios (A1b, A2a, B2a) and present time models. Standard deviation values are given in parenthesis.
(DOC)

S1 Fig Global distribution of the suitable niches of *D*. *majalis* ssp. *lapponica* (A), *D*. *majalis* ssp. *majalis* (B), and *D*. *majalis* ssp. *traunsteineri* (C) in the LGM (PMIP2 based models). (TIF)

S2 Fig Global distribution of the suitable niches of *D*. *incarnata* var. *cruenta* (A), *D*. *incarnata* var. *incarnata* (B), and *D*. *incarnata* var. *ochroleuca* (C) in the LGM (PMIP2 based models). (TIF)

S3 Fig Global distribution of the suitable niches of *D*. *maculata* ssp. *fuchsii* (A) and *D*. *maculata* ssp. *maculata* (B) in the LGM (PMIP2 based models). (TIF)

S4 Fig Global distribution of suitable niches of *D*. *majalis* ssp. *lapponica* (A), *D*. *majalis* ssp. *majalis* (B), and *D*. *majalis* ssp. *traunsteineri* (C) in the Last Glacial Maximum (CMIP5 based models). The current coastal line is indicated with a continuous line.
(TIF)

S5 Fig Global distribution of suitable niches of *D*. *incarnata* var. *cruenta* (A), *D*. *incarnata* var. *incarnata* (B), and *D*. *incarnata* var. *ochroleuca* (C) in the Last Glacial Maximum (CMIP5 based models). The current coastal line is indicated with a continuous line.
(TIF)

S6 Fig Global distribution of suitable niches of *D*. *maculata* ssp. *fuchsii* (A) and *D*. *maculata* ssp. *maculata* (B) in the Last Glacial Maximum (CMIP5 based models). The current coastal line is indicated with a continuous line.
(TIF)

S7 Fig Distribution of suitable niches of *D*. *majalis* ssp. *lapponica* (A), *D*. *majalis* ssp. *majalis* (B), and *D*. *majalis* ssp. *traunsteineri* (C) in 2080 modeled for three various climate change scenarios. (TIF)

S8 Fig Distribution of suitable niches of *D*. *incarnata* var. *cruenta* (A), *D*. *incarnata* var. *incarnata* (B), and *D*. *incarnata* var. *ochroleuca* (C) in 2080 modeled for three various climate change scenarios. (TIF)

S9 Fig Distribution of suitable niches of *D*. *maculata* ssp. *fuchsii* (A) and *D*. *maculata* ssp. *maculata* (B) in 2080 modeled for three various climate change scenarios. (TIF)
