# Supplementary material for: Glacial Refugia and Future Habitat Coverage of Selected Dactylorhiza Representatives (Orchidaceae)
Source: PLoS One. 2015 Nov 23;10(11):e0143478. doi: 10.1371/journal.pone.0143478 (PMC4657909; doi:10.1371/journal.pone.0143478)
Supplement: S1 Annex — (DOC) [file pone.0143478.s001.doc]

**S1 Annex.** **List of localities used in the ecological niche modeling.**

| **Species** | **Country** | **Coordinates** |
| --- | --- | --- |
| *D. incarnata var. cruenta* | Estonia | 21,9333333333333; 58,45 |
| *D. incarnata var. cruenta* | Finland | 25,4; 65,2166666666667 |
| *D. incarnata var. cruenta* | Sweden | 18,8; 57,6666666666667 |
| *D. incarnata var. cruenta* | Sweden | 18,7833333333333; 57,55 |
| *D. incarnata var. cruenta* | Sweden | 18,8333333333333; 57,8166666666667 |
| *D. incarnata var. cruenta* | Sweden | 18,3166666666667; 57,3333333333333 |
| *D. incarnata var. cruenta* | Sweden | 18,7833333333333; 57,55 |
| *D. incarnata var. cruenta* | Sweden | 13,7666666666667; 58,3333333333333 |
| *D. incarnata var. cruenta* | Sweden | 15,1; 58,55 |
| *D. incarnata var. cruenta* | Sweden | 15,5666666666667; 58,3666666666667 |
| *D. incarnata var. cruenta* | Sweden | 15,95; 63,6666666666667 |
| *D. incarnata var. cruenta* | Sweden | 15,0333333333333; 63,3166666666667 |
| *D. incarnata var. cruenta* | Sweden | 19,8; 65,0166666666667 |
| *D. maculata ssp. fuchsii* | Poland | 18,5383333333333; 54,4677777777778 |
| *D. maculata ssp. fuchsii* | Poland | 18,5355555555556; 54,4694444444444 |
| *D. maculata ssp. fuchsii* | Poland | 18,3433333333333; 54,8427777777778 |
| *D. maculata ssp. fuchsii* | Poland | 18,3330555555556; 54,835 |
| *D. maculata ssp. fuchsii* | Poland | 17,0291666666667; 54,4272222222222 |
| *D. maculata ssp. fuchsii* | Poland | 16,9944444444444; 54,2705555555556 |
| *D. maculata ssp. fuchsii* | Poland | 16,8938888888889; 54,2380555555556 |
| *D. maculata ssp. fuchsii* | Poland | 17,3822222222222; 54,2508333333333 |
| *D. maculata ssp. fuchsii* | Poland | 16,8894444444444; 54,2672222222222 |
| *D. maculata ssp. fuchsii* | Poland | 16,3325; 53,9272222222222 |
| *D. maculata ssp. fuchsii* | Poland | 16,3069444444444; 54,1138888888889 |
| *D. maculata ssp. fuchsii* | Poland | 16,9094444444444; 54,5886111111111 |
| *D. maculata ssp. fuchsii* | Italy | 9,38333333333333; 44,3166666666667 |
| *D. maculata ssp. fuchsii* | Italy | 11,6666666666667; 45,5666666666667 |
| *D. maculata ssp. fuchsii* | Italy | 11,8166666666667; 46,4833333333333 |
| *D. maculata ssp. fuchsii* | Italy | 11,7833333333333; 46,5333333333333 |
| *D. maculata ssp. fuchsii* | Italy | 11,8166666666667; 46,5333333333333 |
| *D. maculata ssp. fuchsii* | Romania | 23,45; 46,4 |
| *D. maculata ssp. fuchsii* | Romania | 26,5; 46,1333333333333 |
| *D. maculata ssp. fuchsii* | Romania | 25,5833333333333; 46,3166666666667 |
| *D. maculata ssp. fuchsii* | France | 3,35; 44,1833333333333 |
| *D. maculata ssp. fuchsii* | France | 6,28333333333333; 45,05 |
| *D. maculata ssp. fuchsii* | France | 5,6; 44,7833333333333 |
| *D. maculata ssp. fuchsii* | France | 5,55; 44,8666666666667 |
| *D. maculata ssp. fuchsii* | France | 6,45; 45,2 |
| *D. maculata ssp. fuchsii* | France | 6,9; 45,2833333333333 |
| *D. maculata ssp. fuchsii* | Switzerland | 8,35; 46,55 |
| *D. maculata ssp. fuchsii* | Switzerland | 8,41666666666667; 46,5666666666667 |
| *D. maculata ssp. fuchsii* | Switzerland | 9,76666666666667; 46,5833333333333 |
| *D. maculata ssp. fuchsii* | Switzerland | 8,66666666666667; 46,6666666666667 |
| *D. maculata ssp. fuchsii* | Slovenia | 14,45; 45,75 |
| *D. maculata ssp. fuchsii* | Slovenia | 13,8333333333333; 46,1166666666667 |
| *D. maculata ssp. fuchsii* | Austria | 11,1833333333333; 47,3333333333333 |
| *D. maculata ssp. fuchsii* | Austria | 11,1833333333333; 47,3333333333333 |
| *D. maculata ssp. fuchsii* | Austria | 13,6166666666667; 47,0333333333333 |
| *D. maculata ssp. fuchsii* | Austria | 13,4333333333333; 47,6666666666667 |
| *D. maculata ssp. fuchsii* | Austria | 15,65; 47,4333333333333 |
| *D. maculata ssp. fuchsii* | Austria | 15,2333333333333; 47,5333333333333 |
| *D. maculata ssp. fuchsii* | Austria | 15,3333333333333; 47,75 |
| *D. maculata ssp. fuchsii* | Austria | 15,9666666666667; 47,9666666666667 |
| *D. maculata ssp. fuchsii* | Czech Republic | 18,0833333333333; 49,0833333333333 |
| *D. maculata ssp. fuchsii* | Czech Republic | 13,65; 49,1833333333333 |
| *D. maculata ssp. fuchsii* | Slovakia | 20,3833333333333; 48,8666666666667 |
| *D. maculata ssp. fuchsii* | Germany | 9,21666666666667; 49,1333333333333 |
| *D. maculata ssp. fuchsii* | Germany | 9,21666666666667; 49,1333333333333 |
| *D. maculata ssp. fuchsii* | Germany | 8,18333333333333; 49,9333333333333 |
| *D. maculata ssp. fuchsii* | Germany | 9; 51 |
| *D. maculata ssp. fuchsii* | Germany | 14,6166666666667; 51,2666666666667 |
| *D. maculata ssp. fuchsii* | Poland | 16,1833333333333; 50,7333333333333 |
| *D. maculata ssp. fuchsii* | Poland | 17,6166666666667; 50,9666666666667 |
| *D. maculata ssp. fuchsii* | Poland | 20,0333333333333; 49,8 |
| *D. maculata ssp. fuchsii* | Poland | 18,55; 54,4666666666667 |
| *D. maculata ssp. fuchsii* | Poland | 17,8; 54,7166666666667 |
| *D. maculata ssp. fuchsii* | Wales | 4,3; 53,3166666666667 |
| *D. maculata ssp. fuchsii* | England | 0,316666666666667; 51,2333333333333 |
| *D. maculata ssp. fuchsii* | England | 1,08333333333333; 51,2666666666667 |
| *D. maculata ssp. fuchsii* | England | 1,28333333333333; 51,6666666666667 |
| *D. maculata ssp. fuchsii* | England | 0,916666666666667; 52,3666666666667 |
| *D. maculata ssp. fuchsii* | England | 0,583333333333333; 52,5833333333333 |
| *D. maculata ssp. fuchsii* | England | 0,583333333333333; 52,7166666666667 |
| *D. maculata ssp. fuchsii* | England | 2,21666666666667; 53,9833333333333 |
| *D. maculata ssp. fuchsii* | England | 1,03333333333333; 54,1333333333333 |
| *D. maculata ssp. fuchsii* | England | 1,11666666666667; 54,2666666666667 |
| *D. maculata ssp. fuchsii* | Denmark | 11,7833333333333; 54,7 |
| *D. maculata ssp. fuchsii* | Denmark | 11,3833333333333; 54,8 |
| *D. maculata ssp. fuchsii* | Denmark | 11,4833333333333; 54,8333333333333 |
| *D. maculata ssp. fuchsii* | Denmark | 11,3666666666667; 54,8666666666667 |
| *D. maculata ssp. fuchsii* | Denmark | 12,1666666666667; 55,2166666666667 |
| *D. maculata ssp. fuchsii* | Norway | 10,3; 59,8166666666667 |
| *D. maculata ssp. fuchsii* | Norway | 9,85; 59,0166666666667 |
| *D. maculata ssp. fuchsii* | Norway | 10,0333333333333; 59,5833333333333 |
| *D. maculata ssp. fuchsii* | Norway | 8; 59,4333333333333 |
| *D. maculata ssp. fuchsii* | Norway | 6,71666666666667; 62,5166666666667 |
| *D. maculata ssp. fuchsii* | Norway | 12,0833333333333; 64,2333333333333 |
| *D. maculata ssp. fuchsii* | Norway | 14,4833333333333; 67,3333333333333 |
| *D. maculata ssp. fuchsii* | Norway | 18,8333333333333; 69,1333333333333 |
| *D. maculata ssp. fuchsii* | Norway | 22,95; 70,05 |
| *D. maculata ssp. fuchsii* | Sweden | 13,3833333333333; 55,6833333333333 |
| *D. maculata ssp. fuchsii* | Sweden | 13,9666666666667; 55,85 |
| *D. maculata ssp. fuchsii* | Sweden | 13,6; 55,9666666666667 |
| *D. maculata ssp. fuchsii* | Sweden | 16,6166666666667; 56,6 |
| *D. maculata ssp. fuchsii* | Sweden | 16,5333333333333; 56,6333333333333 |
| *D. maculata ssp. fuchsii* | Sweden | 16,55; 56,65 |
| *D. maculata ssp. fuchsii* | Sweden | 16,85; 57,05 |
| *D. maculata ssp. fuchsii* | Sweden | 18,3166666666667; 57,0666666666667 |
| *D. maculata ssp. fuchsii* | Sweden | 18,4166666666667; 57,1166666666667 |
| *D. maculata ssp. fuchsii* | Sweden | 18,3166666666667; 57,35 |
| *D. maculata ssp. fuchsii* | Sweden | 18,7833333333333; 57,55 |
| *D. maculata ssp. fuchsii* | Sweden | 18,8; 57,6666666666667 |
| *D. maculata ssp. fuchsii* | Sweden | 12,9666666666667; 56,4166666666667 |
| *D. maculata ssp. fuchsii* | Sweden | 13,7333333333333; 58,2333333333333 |
| *D. maculata ssp. fuchsii* | Sweden | 14,6833333333333; 58,3166666666667 |
| *D. maculata ssp. fuchsii* | Sweden | 14,65; 58,3166666666667 |
| *D. maculata ssp. fuchsii* | Sweden | 14,6833333333333; 58,3666666666667 |
| *D. maculata ssp. fuchsii* | Sweden | 15,5333333333333; 58,4166666666667 |
| *D. maculata ssp. fuchsii* | Sweden | 17,1166666666667; 58,75 |
| *D. maculata ssp. fuchsii* | Sweden | 14,1166666666667; 59,8166666666667 |
| *D. maculata ssp. fuchsii* | Sweden | 12,8666666666667; 60,0166666666667 |
| *D. maculata ssp. fuchsii* | Sweden | 14,7833333333333; 59,4166666666667 |
| *D. maculata ssp. fuchsii* | Sweden | 14,7833333333333; 59,4333333333333 |
| *D. maculata ssp. fuchsii* | Sweden | 19,35; 59,7166666666667 |
| *D. maculata ssp. fuchsii* | Sweden | 17,95; 60,5166666666667 |
| *D. maculata ssp. fuchsii* | Sweden | 17,45; 60,5666666666667 |
| *D. maculata ssp. fuchsii* | Sweden | 17,6166666666667; 60,6166666666667 |
| *D. maculata ssp. fuchsii* | Sweden | 14,9; 61,15 |
| *D. maculata ssp. fuchsii* | Sweden | 17,35; 60,6166666666667 |
| *D. maculata ssp. fuchsii* | Sweden | 16,75; 61,5333333333333 |
| *D. maculata ssp. fuchsii* | Sweden | 14,9; 61,6166666666667 |
| *D. maculata ssp. fuchsii* | Sweden | 15,7; 62,5666666666667 |
| *D. maculata ssp. fuchsii* | Sweden | 16,4166666666667; 63,5166666666667 |
| *D. maculata ssp. fuchsii* | Sweden | 15,9833333333333; 63,6166666666667 |
| *D. maculata ssp. fuchsii* | Sweden | 12,25; 62,5666666666667 |
| *D. maculata ssp. fuchsii* | Sweden | 14,6333333333333; 63,2333333333333 |
| *D. maculata ssp. fuchsii* | Sweden | 15,3333333333333; 63,4666666666667 |
| *D. maculata ssp. fuchsii* | Sweden | 17,95; 64,85 |
| *D. maculata ssp. fuchsii* | Sweden | 18,7833333333333; 68,3333333333333 |
| *D. maculata ssp. fuchsii* | Estonia | 22,1333333333333; 58,2833333333333 |
| *D. maculata ssp. fuchsii* | Estonia | 21,9666666666667; 58,3333333333333 |
| *D. maculata ssp. fuchsii* | Estonia | 21,9666666666667; 58,4333333333333 |
| *D. maculata ssp. fuchsii* | Estonia | 26,4833333333333; 58,0666666666667 |
| *D. maculata ssp. fuchsii* | Estonia | 26,4833333333333; 57,7166666666667 |
| *D. maculata ssp. fuchsii* | Estonia | 23,5166666666667; 58,5666666666667 |
| *D. maculata ssp. fuchsii* | Estonia | 23,55; 58,5666666666667 |
| *D. maculata ssp. fuchsii* | Estonia | 23,8166666666667; 58,7166666666667 |
| *D. maculata ssp. fuchsii* | Estonia | 26,2666666666667; 58,5833333333333 |
| *D. maculata ssp. fuchsii* | Russia | 34,75; 60,3166666666667 |
| *D. maculata ssp. fuchsii* | Russia | 34,7666666666667; 60,3166666666667 |
| *D. maculata ssp. fuchsii* | Russia | 31,3666666666667; 61,6666666666667 |
| *D. maculata ssp. fuchsii* | Russia | 30,15; 66,7833333333333 |
| *D. maculata ssp. fuchsii* | Russia | 59,85; 56,8166666666667 |
| *D. maculata ssp. fuchsii* | Russia | 59,9166666666667; 60,5166666666667 |
| *D. maculata ssp. fuchsii* | Belgium | 2,68777777777778; 51,1341666666667 |
| *D. maculata ssp. fuchsii* | Belgium | 2,98444444444444; 51,2586111111111 |
| *D. maculata ssp. fuchsii* | Belgium | 5,19166666666667; 50,1319444444444 |
| *D. maculata ssp. fuchsii* | Belgium | 4,79583333333333; 50,2927777777778 |
| *D. maculata ssp. fuchsii* | France | 4,53305555555556; 48,2663888888889 |
| *D. maculata ssp. fuchsii* | France | 3,07138888888889; 43,8780555555556 |
| *D. maculata ssp. fuchsii* | France | 3,12138888888889; 43,8619444444444 |
| *D. maculata ssp. fuchsii* | Italy | 9,49166666666667; 44,3833333333333 |
| *D. maculata ssp. fuchsii* | Poland | 18,1975; 54,7513888888889 |
| *D. maculata ssp. fuchsii* | Poland | 18,2497222222222; 54,6008333333333 |
| *D. maculata ssp. fuchsii* | Poland | 20,4244444444444; 49,4266666666667 |
| *D. maculata ssp. fuchsii* | Poland | 20,3577777777778; 49,4347222222222 |
| *D. incarnata var. incarnata* | Poland | 17,8; 54,2263888888889 |
| *D. incarnata var. incarnata* | Poland | 17,8413888888889; 53,9255555555556 |
| *D. incarnata var. incarnata* | Poland | 18,5163888888889; 54,63 |
| *D. incarnata var. incarnata* | Poland | 17,0019444444444; 54,1830555555556 |
| *D. incarnata var. incarnata* | Poland | 17,5491666666667; 53,9791666666667 |
| *D. incarnata var. incarnata* | Poland | 17,3608333333333; 54,1966666666667 |
| *D. incarnata var. incarnata* | Poland | 17,5136111111111; 54,2594444444444 |
| *D. incarnata var. incarnata* | Poland | 16,3066666666667; 54,1158333333333 |
| *D. incarnata var. incarnata* | Poland | 18,4791666666667; 54,3175 |
| *D. incarnata var. incarnata* | Poland | 18,3019444444444; 54,8269444444444 |
| *D. incarnata var. incarnata* | Poland | 14,5283333333333; 53,4786111111111 |
| *D. incarnata var. incarnata* | Poland | 14,92; 53,2116666666667 |
| *D. incarnata var. incarnata* | Poland | 14,8694444444444; 53,4922222222222 |
| *D. incarnata var. incarnata* | Poland | 22,1447222222222; 54,1319444444444 |
| *D. incarnata var. incarnata* | Poland | 22,0147222222222; 54,2413888888889 |
| *D. incarnata var. incarnata* | Poland | 22,1388888888889; 54,2244444444444 |
| *D. incarnata var. incarnata* | Poland | 22,8344444444444; 53,4888888888889 |
| *D. incarnata var. incarnata* | Denmark | 8,95611111111111; 57,0286111111111 |
| *D. incarnata var. incarnata* | Denmark | 8,86666666666667; 56,9458333333333 |
| *D. incarnata var. incarnata* | Denmark | 10,5133333333333; 56,0994444444444 |
| *D. incarnata var. incarnata* | Denmark | 12,415; 56,0808333333333 |
| *D. incarnata var. incarnata* | Denmark | 11,2975; 55,7375 |
| *D. incarnata var. incarnata* | Denmark | 12,1852777777778; 55,6461111111111 |
| *D. incarnata var. incarnata* | Denmark | 12,2244444444444; 55,4247222222222 |
| *D. incarnata var. incarnata* | Denmark | 12,2616666666667; 55,39 |
| *D. incarnata var. incarnata* | Wales | 4,13777777777778; 52,8086111111111 |
| *D. incarnata var. incarnata* | Wales | 4,12388888888889; 52,8619444444444 |
| *D. incarnata var. incarnata* | Wales | 4,05111111111111; 52,5252777777778 |
| *D. incarnata var. incarnata* | Denmark | 9,88138888888889; 57,5180555555556 |
| *D. incarnata var. incarnata* | Denmark | 9,86916666666667; 57,5144444444444 |
| *D. incarnata var. incarnata* | Denmark | 9,28; 57,1419444444444 |
| *D. incarnata var. incarnata* | Netherlands | 4,03833333333333; 51,8927777777778 |
| *D. incarnata var. incarnata* | Netherlands | 4,06277777777778; 51,9166666666667 |
| *D. incarnata var. incarnata* | Netherlands | 4,04333333333333; 51,9261111111111 |
| *D. incarnata var. incarnata* | Belgium | 2,68777777777778; 51,1341666666667 |
| *D. incarnata var. incarnata* | Belgium | 2,98444444444444; 51,2586111111111 |
| *D. incarnata var. incarnata* | Estonia | 21,9666666666667; 58,4333333333333 |
| *D. incarnata var. incarnata* | Estonia | 22; 58,3166666666667 |
| *D. incarnata var. incarnata* | Estonia | 22,4666666666667; 58,9833333333333 |
| *D. incarnata var. incarnata* | Estonia | 23,5166666666667; 58,5666666666667 |
| *D. incarnata var. incarnata* | Finland | 25,7333333333333; 62,2333333333333 |
| *D. incarnata var. incarnata* | Finland | 25,4; 65,2166666666667 |
| *D. incarnata var. incarnata* | Finland | 26,8666666666667; 67,5666666666667 |
| *D. incarnata var. incarnata* | Norway | 10,0666666666667; 59,7166666666667 |
| *D. incarnata var. incarnata* | Norway | 10,3; 59,8166666666667 |
| *D. incarnata var. incarnata* | Russia | 33,8; 61,7833333333333 |
| *D. incarnata var. incarnata* | Russia | 34; 62,1166666666667 |
| *D. incarnata var. incarnata* | Russia | 33,3833333333333; 67,5666666666667 |
| *D. incarnata var. incarnata* | Sweden | 18,8; 57,6666666666667 |
| *D. incarnata var. incarnata* | Sweden | 18,7833333333333; 57,55 |
| *D. incarnata var. incarnata* | Sweden | 18,7; 57,9166666666667 |
| *D. incarnata var. incarnata* | Sweden | 18,8333333333333; 57,8166666666667 |
| *D. incarnata var. incarnata* | Sweden | 18,2; 56,9166666666667 |
| *D. incarnata var. incarnata* | Sweden | 18,3166666666667; 57,3333333333333 |
| *D. incarnata var. incarnata* | Sweden | 18,2; 56,9333333333333 |
| *D. incarnata var. incarnata* | Sweden | 18,7833333333333; 57,55 |
| *D. incarnata var. incarnata* | Sweden | 13,8; 58,1666666666667 |
| *D. incarnata var. incarnata* | Sweden | 13,7666666666667; 58,3333333333333 |
| *D. incarnata var. incarnata* | Sweden | 14,7666666666667; 59,4 |
| *D. incarnata var. incarnata* | Sweden | 14,95; 58,4666666666667 |
| *D. incarnata var. incarnata* | Sweden | 15,1; 58,55 |
| *D. incarnata var. incarnata* | Sweden | 15,5666666666667; 58,3666666666667 |
| *D. incarnata var. incarnata* | Sweden | 14,9; 61,6166666666667 |
| *D. incarnata var. incarnata* | Sweden | 15,95; 63,6666666666667 |
| *D. incarnata var. incarnata* | Sweden | 14,0333333333333; 63,85 |
| *D. incarnata var. incarnata* | Sweden | 19,8; 65,0166666666667 |
| *D. incarnata var. incarnata* | Belgium | 5,53527777777778; 49,6944444444444 |
| *D. incarnata var. incarnata* | Belgium | 5,58111111111111; 49,5519444444444 |
| *D. incarnata var. incarnata* | France | 4,53305555555556; 48,2663888888889 |
| *D. incarnata var. incarnata* | Italy | 9,49166666666667; 44,3833333333333 |
| *D. incarnata var. incarnata* | Poland | 18,1333333333333; 54,6166666666667 |
| *D. incarnata var. incarnata* | Poland | 18,4366666666667; 54,0988888888889 |
| *D. incarnata var. incarnata* | Poland | 18,5541666666667; 53,6633333333333 |
| *D. incarnata var. incarnata* | Poland | 22,5825; 53,2838888888889 |
| *D. incarnata var. incarnata* | Finland | 26,2; 62,05 |
| *D. incarnata var. incarnata* | Finland | 25,7333333333333; 62,2333333333333 |
| *D. majalis ssp. lapponica* | Norway | 9,06666666666667; 63,05 |
| *D. majalis ssp. lapponica* | Norway | 11,8333333333333; 62,6833333333333 |
| *D. majalis ssp. lapponica* | Norway | 11,8333333333333; 62,6666666666667 |
| *D. majalis ssp. lapponica* | Sweden | 15,5333333333333; 65,7333333333333 |
| *D. majalis ssp. lapponica* | Sweden | 15,0833333333333; 65,7 |
| *D. majalis ssp. lapponica* | Norway | 11,3833333333333; 62,5666666666667 |
| *D. majalis ssp. lapponica* | Sweden | 15,35; 64,7333333333333 |
| *D. majalis ssp. lapponica* | Sweden | 12,25; 62,5666666666667 |
| *D. majalis ssp. lapponica* | Sweden | 12,3; 62,7166666666667 |
| *D. majalis ssp. lapponica* | Sweden | 14,45; 62,5666666666667 |
| *D. majalis ssp. lapponica* | Sweden | 15,3333333333333; 63,4666666666667 |
| *D. majalis ssp. lapponica* | Sweden | 15,1666666666667; 65,6833333333333 |
| *D. majalis ssp. lapponica* | Sweden | 23,2166666666667; 67,3333333333333 |
| *D. majalis ssp. lapponica* | Sweden | 15,5; 65,75 |
| *D. majalis ssp. lapponica* | Sweden | 15,1166666666667; 65,7 |
| *D. majalis ssp. lapponica* | Sweden | 23,5; 66,3166666666667 |
| *D. majalis ssp. lapponica* | Sweden | 19,5666666666667; 64,9833333333333 |
| *D. majalis ssp. lapponica* | Sweden | 14,95; 65,75 |
| *D. majalis ssp. lapponica* | Sweden | 18,8666666666667; 59,7 |
| *D. majalis ssp. lapponica* | Sweden | 18,2333333333333; 59,8666666666667 |
| *D. majalis ssp. lapponica* | Sweden | 18,35; 60,0166666666667 |
| *D. majalis ssp. lapponica* | Sweden | 18,3166666666667; 57,35 |
| *D. majalis ssp. lapponica* | Sweden | 18,6833333333333; 57,7 |
| *D. majalis ssp. lapponica* | Sweden | 18,9; 57,8166666666667 |
| *D. majalis ssp. lapponica* | Sweden | 18,8833333333333; 59,7166666666667 |
| *D. majalis ssp. lapponica* | Sweden | 18,3666666666667; 60,0333333333333 |
| *D. maculata ssp. maculata* | Poland | 18,5355555555556; 54,4680555555556 |
| *D. maculata ssp. maculata* | Poland | 18,0588888888889; 54,8275 |
| *D. maculata ssp. maculata* | Poland | 17,4269444444444; 54,3594444444444 |
| *D. maculata ssp. maculata* | Poland | 16,8938888888889; 54,2377777777778 |
| *D. maculata ssp. maculata* | Poland | 14,8694444444444; 53,4922222222222 |
| *D. maculata ssp. maculata* | Poland | 14,4461111111111; 53,6141666666667 |
| *D. maculata ssp. maculata* | Poland | 22,1608333333333; 54,2322222222222 |
| *D. maculata ssp. maculata* | Poland | 22,1505555555556; 54,1430555555556 |
| *D. maculata ssp. maculata* | Finland | 26,3333333333333; 62,6666666666667 |
| *D. maculata ssp. maculata* | Portugal | 8,18333333333333; 40,5666666666667 |
| *D. maculata ssp. maculata* | Portugal | 7,58333333333333; 41,7166666666667 |
| *D. maculata ssp. maculata* | Portugal | 7,95; 41,7333333333333 |
| *D. maculata ssp. maculata* | Portugal | 7,61666666666667; 41,75 |
| *D. maculata ssp. maculata* | Portugal | 7,68333333333333; 41,75 |
| *D. maculata ssp. maculata* | Portugal | 7,83333333333333; 41,8166666666667 |
| *D. maculata ssp. maculata* | Romania | 25,7666666666667; 46,2333333333333 |
| *D. maculata ssp. maculata* | Romania | 25,5833333333333; 46,3166666666667 |
| *D. maculata ssp. maculata* | Romania | 25,6833333333333; 46,3666666666667 |
| *D. maculata ssp. maculata* | France | 5,18333333333333; 44,8666666666667 |
| *D. maculata ssp. maculata* | France | 6,51666666666667; 45,7666666666667 |
| *D. maculata ssp. maculata* | Slovenia | 14,45; 45,75 |
| *D. maculata ssp. maculata* | Slovenia | 14,4833333333333; 45,7833333333333 |
| *D. maculata ssp. maculata* | Slovenia | 13,8333333333333; 46,1166666666667 |
| *D. maculata ssp. maculata* | Czech Republic | 13,8; 50,7166666666667 |
| *D. maculata ssp. maculata* | Czech Republic | 14,6; 50,6 |
| *D. maculata ssp. maculata* | Netherlands | 3,76666666666667; 51,55 |
| *D. maculata ssp. maculata* | Netherlands | 6,03333333333333; 50,9333333333333 |
| *D. maculata ssp. maculata* | Netherlands | 5,33333333333333; 53,4 |
| *D. maculata ssp. maculata* | Poland | 16,7666666666667; 50,5666666666667 |
| *D. maculata ssp. maculata* | Poland | 16,7333333333333; 50,85 |
| *D. maculata ssp. maculata* | Poland | 19,7; 49,4166666666667 |
| *D. maculata ssp. maculata* | Poland | 17,75; 51,85 |
| *D. maculata ssp. maculata* | Poland | 17,8833333333333; 54,1166666666667 |
| *D. maculata ssp. maculata* | Poland | 18,1666666666667; 54,65 |
| *D. maculata ssp. maculata* | Poland | 18,0666666666667; 54,8166666666667 |
| *D. maculata ssp. maculata* | Wales | 4,33333333333333; 53,2666666666667 |
| *D. maculata ssp. maculata* | Wales | 4,3; 53,3166666666667 |
| *D. maculata ssp. maculata* | Wales | 3,85; 53,3333333333333 |
| *D. maculata ssp. maculata* | England | 1,58333333333333; 50,8666666666667 |
| *D. maculata ssp. maculata* | England | 0,716666666666667; 51,1333333333333 |
| *D. maculata ssp. maculata* | England | 2,01666666666667; 54,0833333333333 |
| *D. maculata ssp. maculata* | England | 1,11666666666667; 54,2666666666667 |
| *D. maculata ssp. maculata* | Faroe Islands | 6,76666666666667; 62,05 |
| *D. maculata ssp. maculata* | Faroe Islands | 7,16666666666667; 62,15 |
| *D. maculata ssp. maculata* | Faroe Islands | 7,08333333333333; 62,3 |
| *D. maculata ssp. maculata* | Iceland | 20,2333333333333; 63,75 |
| *D. maculata ssp. maculata* | Iceland | 22,0833333333333; 63,95 |
| *D. maculata ssp. maculata* | Iceland | 22,0666666666667; 63,9666666666667 |
| *D. maculata ssp. maculata* | Iceland | 21,5333333333333; 64,1166666666667 |
| *D. maculata ssp. maculata* | Iceland | 16; 64,1666666666667 |
| *D. maculata ssp. maculata* | Iceland | 14,1833333333333; 65,0333333333333 |
| *D. maculata ssp. maculata* | Iceland | 13,5833333333333; 66,1166666666667 |
| *D. maculata ssp. maculata* | Denmark | 9,71666666666667; 56,0833333333333 |
| *D. maculata ssp. maculata* | Norway | 10,85; 59,7666666666667 |
| *D. maculata ssp. maculata* | Norway | 10,1; 59,7166666666667 |
| *D. maculata ssp. maculata* | Norway | 10,1666666666667; 59,5 |
| *D. maculata ssp. maculata* | Norway | 9,5; 59,2666666666667 |
| *D. maculata ssp. maculata* | Norway | 5,6; 58,8833333333333 |
| *D. maculata ssp. maculata* | Norway | 5,58333333333333; 59,0333333333333 |
| *D. maculata ssp. maculata* | Norway | 5,33333333333333; 60,3333333333333 |
| *D. maculata ssp. maculata* | Norway | 4,86666666666667; 60,7833333333333 |
| *D. maculata ssp. maculata* | Norway | 4,81666666666667; 60,8 |
| *D. maculata ssp. maculata* | Norway | 5,86666666666667; 61,45 |
| *D. maculata ssp. maculata* | Norway | 6,83333333333333; 62,4833333333333 |
| *D. maculata ssp. maculata* | Norway | 6,1; 62,5666666666667 |
| *D. maculata ssp. maculata* | Norway | 8,16666666666667; 63,1666666666667 |
| *D. maculata ssp. maculata* | Norway | 13,1166666666667; 64,4833333333333 |
| *D. maculata ssp. maculata* | Norway | 14,1833333333333; 65,8333333333333 |
| *D. maculata ssp. maculata* | Norway | 15,3333333333333; 66,8333333333333 |
| *D. maculata ssp. maculata* | Norway | 15,5333333333333; 67,4833333333333 |
| *D. maculata ssp. maculata* | Norway | 16,2666666666667; 68,2333333333333 |
| *D. maculata ssp. maculata* | Norway | 22,05; 70,0666666666667 |
| *D. maculata ssp. maculata* | Norway | 30,0166666666667; 69,7 |
| *D. maculata ssp. maculata* | Norway | 29,45; 70,1 |
| *D. maculata ssp. maculata* | Norway | 28,1; 70,4166666666667 |
| *D. maculata ssp. maculata* | Norway | 26,8333333333333; 70,45 |
| *D. maculata ssp. maculata* | Sweden | 14,2666666666667; 55,2833333333333 |
| *D. maculata ssp. maculata* | Sweden | 13,2833333333333; 55,7166666666667 |
| *D. maculata ssp. maculata* | Sweden | 14,0666666666667; 55,9833333333333 |
| *D. maculata ssp. maculata* | Sweden | 14,45; 56,0166666666667 |
| *D. maculata ssp. maculata* | Sweden | 13,9666666666667; 56,1 |
| *D. maculata ssp. maculata* | Sweden | 14,0666666666667; 56,2333333333333 |
| *D. maculata ssp. maculata* | Sweden | 13,2; 56,2666666666667 |
| *D. maculata ssp. maculata* | Sweden | 13,3; 56,2833333333333 |
| *D. maculata ssp. maculata* | Sweden | 13,0666666666667; 56,3166666666667 |
| *D. maculata ssp. maculata* | Sweden | 14,1166666666667; 56,4333333333333 |
| *D. maculata ssp. maculata* | Sweden | 13,95; 56,45 |
| *D. maculata ssp. maculata* | Sweden | 15,5333333333333; 56,25 |
| *D. maculata ssp. maculata* | Sweden | 16,4166666666667; 56,2333333333333 |
| *D. maculata ssp. maculata* | Sweden | 18,3166666666667; 57,35 |
| *D. maculata ssp. maculata* | Sweden | 18,3833333333333; 57,5833333333333 |
| *D. maculata ssp. maculata* | Sweden | 18,8; 57,7166666666667 |
| *D. maculata ssp. maculata* | Sweden | 16,35; 56,6666666666667 |
| *D. maculata ssp. maculata* | Sweden | 13,7333333333333; 56,7666666666667 |
| *D. maculata ssp. maculata* | Sweden | 14,0166666666667; 57,7833333333333 |
| *D. maculata ssp. maculata* | Sweden | 13,15; 56,75 |
| *D. maculata ssp. maculata* | Sweden | 13,1666666666667; 56,8166666666667 |
| *D. maculata ssp. maculata* | Sweden | 12,3833333333333; 58,9 |
| *D. maculata ssp. maculata* | Sweden | 12,4833333333333; 59,05 |
| *D. maculata ssp. maculata* | Sweden | 12,4666666666667; 59,0666666666667 |
| *D. maculata ssp. maculata* | Sweden | 13,9333333333333; 57,85 |
| *D. maculata ssp. maculata* | Sweden | 13,85; 58,4333333333333 |
| *D. maculata ssp. maculata* | Sweden | 14,5166666666667; 58,6333333333333 |
| *D. maculata ssp. maculata* | Sweden | 15,5166666666667; 57,95 |
| *D. maculata ssp. maculata* | Sweden | 15,5666666666667; 58,3666666666667 |
| *D. maculata ssp. maculata* | Sweden | 15,1166666666667; 58,8666666666667 |
| *D. maculata ssp. maculata* | Sweden | 14,7166666666667; 58,9166666666667 |
| *D. maculata ssp. maculata* | Sweden | 17,1166666666667; 58,75 |
| *D. maculata ssp. maculata* | Sweden | 16,8166666666667; 58,9666666666667 |
| *D. maculata ssp. maculata* | Sweden | 12,9; 59,1333333333333 |
| *D. maculata ssp. maculata* | Sweden | 14,1; 59,8333333333333 |
| *D. maculata ssp. maculata* | Sweden | 12,8666666666667; 60,0166666666667 |
| *D. maculata ssp. maculata* | Sweden | 14,7833333333333; 59,4333333333333 |
| *D. maculata ssp. maculata* | Sweden | 14,8166666666667; 59,4833333333333 |
| *D. maculata ssp. maculata* | Sweden | 14,9666666666667; 59,9833333333333 |
| *D. maculata ssp. maculata* | Sweden | 14,8333333333333; 60,0333333333333 |
| *D. maculata ssp. maculata* | Sweden | 18,5666666666667; 59,25 |
| *D. maculata ssp. maculata* | Sweden | 18,6666666666667; 59,8833333333333 |
| *D. maculata ssp. maculata* | Sweden | 18,35; 60,0333333333333 |
| *D. maculata ssp. maculata* | Sweden | 18,3166666666667; 60,2833333333333 |
| *D. maculata ssp. maculata* | Sweden | 17,3833333333333; 60,4 |
| *D. maculata ssp. maculata* | Sweden | 15,6; 59,9666666666667 |
| *D. maculata ssp. maculata* | Sweden | 17,35; 60,6166666666667 |
| *D. maculata ssp. maculata* | Sweden | 17,1333333333333; 61,05 |
| *D. maculata ssp. maculata* | Sweden | 16,7; 61,2 |
| *D. maculata ssp. maculata* | Sweden | 16,8666666666667; 61,2666666666667 |
| *D. maculata ssp. maculata* | Sweden | 16,8333333333333; 61,4666666666667 |
| *D. maculata ssp. maculata* | Sweden | 16,7333333333333; 61,5333333333333 |
| *D. maculata ssp. maculata* | Sweden | 14,9; 61,6166666666667 |
| *D. maculata ssp. maculata* | Sweden | 17,45; 62,25 |
| *D. maculata ssp. maculata* | Sweden | 17,3; 62,3833333333333 |
| *D. maculata ssp. maculata* | Sweden | 18,5166666666667; 62,9833333333333 |
| *D. maculata ssp. maculata* | Sweden | 16,7666666666667; 63,3333333333333 |
| *D. maculata ssp. maculata* | Sweden | 16,4166666666667; 63,5166666666667 |
| *D. maculata ssp. maculata* | Sweden | 12,25; 62,5666666666667 |
| *D. maculata ssp. maculata* | Sweden | 12,3; 62,7166666666667 |
| *D. maculata ssp. maculata* | Sweden | 14,45; 62,5666666666667 |
| *D. maculata ssp. maculata* | Sweden | 20,45; 64,9333333333333 |
| *D. maculata ssp. maculata* | Sweden | 19,5666666666667; 64,9833333333333 |
| *D. maculata ssp. maculata* | Sweden | 23,2333333333333; 67,2833333333333 |
| *D. maculata ssp. maculata* | Sweden | 23,2166666666667; 67,3333333333333 |
| *D. maculata ssp. maculata* | Sweden | 15,35; 64,7 |
| *D. maculata ssp. maculata* | Sweden | 15,35; 64,7333333333333 |
| *D. maculata ssp. maculata* | Sweden | 15,1333333333333; 65,2333333333333 |
| *D. maculata ssp. maculata* | Sweden | 15,1166666666667; 65,6833333333333 |
| *D. maculata ssp. maculata* | Sweden | 15,1166666666667; 65,7 |
| *D. maculata ssp. maculata* | Sweden | 15,0833333333333; 65,75 |
| *D. maculata ssp. maculata* | Sweden | 18,5833333333333; 68,4166666666667 |
| *D. maculata ssp. maculata* | Estonia | 22,1333333333333; 58,2833333333333 |
| *D. maculata ssp. maculata* | Estonia | 22,2833333333333; 58,9333333333333 |
| *D. maculata ssp. maculata* | Estonia | 23,8166666666667; 58,5666666666667 |
| *D. maculata ssp. maculata* | Finland | 20,05; 60,2833333333333 |
| *D. maculata ssp. maculata* | Finland | 26,2; 62,5 |
| *D. maculata ssp. maculata* | Finland | 29,9; 64,7833333333333 |
| *D. maculata ssp. maculata* | Finland | 25,4; 65,2166666666667 |
| *D. maculata ssp. maculata* | Finland | 25; 66,0833333333333 |
| *D. maculata ssp. maculata* | Finland | 24,9; 67,4833333333333 |
| *D. maculata ssp. maculata* | Finland | 26,8666666666667; 67,5666666666667 |
| *D. maculata ssp. maculata* | Russia | 34,7666666666667; 60,3166666666667 |
| *D. maculata ssp. maculata* | Russia | 29,2833333333333; 60,6166666666667 |
| *D. maculata ssp. maculata* | Russia | 34,4666666666667; 61,7333333333333 |
| *D. maculata ssp. maculata* | Russia | 33,8; 61,7833333333333 |
| *D. maculata ssp. maculata* | Russia | 30,15; 66,7833333333333 |
| *D. maculata ssp. maculata* | Russia | 33,3833333333333; 67,5666666666667 |
| *D. maculata ssp. maculata* | Russia | 60,45; 56,95 |
| *D. maculata ssp. maculata* | Russia | 60,15; 57,1833333333333 |
| *D. maculata ssp. maculata* | Denmark | 12,415; 56,0808333333333 |
| *D. maculata ssp. maculata* | Denmark | 11,9272222222222; 55,8969444444444 |
| *D. maculata ssp. maculata* | Denmark | 11,7505555555556; 55,7566666666667 |
| *D. maculata ssp. maculata* | Denmark | 11,4341666666667; 55,8277777777778 |
| *D. maculata ssp. maculata* | Denmark | 9,18722222222222; 56,5175 |
| *D. maculata ssp. maculata* | Denmark | 9,3825; 57,1197222222222 |
| *D. maculata ssp. maculata* | Denmark | 9,83; 57,4591666666667 |
| *D. maculata ssp. maculata* | Denmark | 9,94972222222222; 57,5808333333333 |
| *D. maculata ssp. maculata* | Denmark | 10,3452777777778; 57,5586111111111 |
| *D. maculata ssp. maculata* | Belgium | 5,71666666666667; 49,6630555555556 |
| *D. maculata ssp. maculata* | Belgium | 5,675; 50,3736111111111 |
| *D. maculata ssp. maculata* | France | 3,66666666666667; 48,4833333333333 |
| *D. maculata ssp. maculata* | France | 4,4; 48,6305555555556 |
| *D. maculata ssp. maculata* | Poland | 20,4044444444444; 49,42 |
| *D. majalis ssp. majalis* | Poland | 17,7786111111111; 54,2291666666667 |
| *D. majalis ssp. majalis* | Poland | 18,5175; 54,6227777777778 |
| *D. majalis ssp. majalis* | Poland | 18,4172222222222; 54,7888888888889 |
| *D. majalis ssp. majalis* | Poland | 18,3044444444444; 54,8086111111111 |
| *D. majalis ssp. majalis* | Poland | 18,3019444444444; 54,8269444444444 |
| *D. majalis ssp. majalis* | Poland | 18,5355555555556; 54,4680555555556 |
| *D. majalis ssp. majalis* | Poland | 18,1633333333333; 54,6563888888889 |
| *D. majalis ssp. majalis* | Poland | 18,1691666666667; 54,6594444444444 |
| *D. majalis ssp. majalis* | Poland | 18,1516666666667; 54,6744444444444 |
| *D. majalis ssp. majalis* | Poland | 18,3241666666667; 54,4736111111111 |
| *D. majalis ssp. majalis* | Poland | 18,4211111111111; 54,5275 |
| *D. majalis ssp. majalis* | Poland | 18,3269444444444; 54,5216666666667 |
| *D. majalis ssp. majalis* | Poland | 18,3425; 54,5141666666667 |
| *D. majalis ssp. majalis* | Poland | 18,4661111111111; 54,6616666666667 |
| *D. majalis ssp. majalis* | Poland | 18,4894444444444; 54,4602777777778 |
| *D. majalis ssp. majalis* | Poland | 18,4791666666667; 54,4063888888889 |
| *D. majalis ssp. majalis* | Poland | 17,8397222222222; 53,9263888888889 |
| *D. majalis ssp. majalis* | Poland | 17,06; 54,2497222222222 |
| *D. majalis ssp. majalis* | Poland | 17,4283333333333; 54,3555555555556 |
| *D. majalis ssp. majalis* | Poland | 17,0236111111111; 54,3422222222222 |
| *D. majalis ssp. majalis* | Poland | 16,7372222222222; 54,0602777777778 |
| *D. majalis ssp. majalis* | Poland | 17,5086111111111; 54,3744444444444 |
| *D. majalis ssp. majalis* | Poland | 18,08; 54,2394444444444 |
| *D. majalis ssp. majalis* | Poland | 15,9494444444444; 54,0011111111111 |
| *D. majalis ssp. majalis* | Poland | 14,5363888888889; 53,4808333333333 |
| *D. majalis ssp. majalis* | Poland | 14,8694444444444; 53,4922222222222 |
| *D. majalis ssp. majalis* | Poland | 14,48; 52,9563888888889 |
| *D. majalis ssp. majalis* | Denmark | 11,8433333333333; 55,7052777777778 |
| *D. majalis ssp. majalis* | Denmark | 11,7761111111111; 55,1536111111111 |
| *D. majalis ssp. majalis* | Denmark | 11,8163888888889; 55,1441666666667 |
| *D. majalis ssp. majalis* | Denmark | 12,2455555555556; 55,8194444444444 |
| *D. majalis ssp. majalis* | Denmark | 12,2616666666667; 55,39 |
| *D. majalis ssp. majalis* | Denmark | 10,0472222222222; 56,1802777777778 |
| *D. majalis ssp. majalis* | Denmark | 10,5133333333333; 56,0994444444444 |
| *D. majalis ssp. majalis* | Belgium | 5,05333333333333; 50,1758333333333 |
| *D. majalis ssp. majalis* | Belgium | 4,27083333333333; 50,6625 |
| *D. majalis ssp. majalis* | Belgium | 5,53527777777778; 49,6944444444444 |
| *D. majalis ssp. majalis* | Belgium | 5,25833333333333; 50,1105555555556 |
| *D. majalis ssp. majalis* | Belgium | 5,675; 50,3736111111111 |
| *D. majalis ssp. majalis* | Poland | 20,41; 49,4222222222222 |
| *D. majalis ssp. majalis* | Poland | 20,4088888888889; 49,4197222222222 |
| *D. majalis ssp. majalis* | Sweden | 14,1166666666667; 55,7666666666667 |
| *D. majalis ssp. majalis* | Sweden | 13,9166666666667; 55,5 |
| *D. majalis ssp. majalis* | Sweden | 12,9333333333333; 55,8166666666667 |
| *D. majalis ssp. majalis* | Sweden | 14,1166666666667; 55,7666666666667 |
| *D. majalis ssp. majalis* | Sweden | 13,9166666666667; 55,5 |
| *D. majalis ssp. majalis* | Sweden | 12,9333333333333; 55,8166666666667 |
| *D. majalis ssp. majalis* | Poland | 20,4077777777778; 49,4225 |
| *D. majalis ssp. majalis* | Poland | 20,4091666666667; 49,4213888888889 |
| *D. majalis ssp. majalis* | Poland | 20,3975; 49,4180555555556 |
| *D. majalis ssp. majalis* | Poland | 20,3733333333333; 49,4163888888889 |
| *D. majalis ssp. majalis* | Poland | 20,3386111111111; 49,4377777777778 |
| *Dactylorhiza occidentalis* | Ireland | 9,28333333333333; 53,0166666666667 |
| *D. incarnata var. ochroleuca* | Sweden | 18,8333333333333; 57,8166666666667 |
| *D. incarnata var. ochroleuca* | Sweden | 18,3166666666667; 57,3333333333333 |
| *D. incarnata var. ochroleuca* | Sweden | 18,7833333333333; 57,55 |
| *D. incarnata var. ochroleuca* | Sweden | 13,7666666666667; 58,3333333333333 |
| *D. incarnata var. ochroleuca* | Sweden | 15,1; 58,55 |
| *D. incarnata var. ochroleuca* | Poland | 22,5825; 53,2838888888889 |
| *D. incarnata var. ochroleuca* | Estonia | 21,9666666666667; 58,4333333333333 |
| *D. incarnata var. ochroleuca* | Sweden | 13,9666666666667; 56,1 |
| *D. incarnata var. ochroleuca* | Sweden | 18,7; 57,9166666666667 |
| *D. incarnata var. ochroleuca* | Sweden | 18,2; 56,9166666666667 |
| *D. incarnata var. ochroleuca* | Sweden | 18,2; 56,9333333333333 |
| *D. incarnata var. ochroleuca* | Sweden | 13,8; 58,1666666666667 |
| *D. incarnata var. ochroleuca* | Sweden | 13,85; 58,4333333333333 |
| *D. incarnata var. ochroleuca* | Sweden | 13,7666666666667; 58,3333333333333 |
| *D. incarnata var. ochroleuca* | Sweden | 14,95; 58,4666666666667 |
| *D. incarnata var. ochroleuca* | Sweden | 15,05; 58,4166666666667 |
| *D. incarnata var. ochroleuca* | Sweden | 15,0333333333333; 63,3166666666667 |
| *D. majalis ssp. traunsteineri* | Sweden | 18,8833333333333; 57,8166666666667 |
| *D. majalis ssp. traunsteineri* | Sweden | 18,3166666666667; 57,3333333333333 |
| *D. majalis ssp. traunsteineri* | Sweden | 18,8; 57,85 |
| *D. majalis ssp. traunsteineri* | Sweden | 15,5333333333333; 58,4166666666667 |
| *D. majalis ssp. traunsteineri* | Sweden | 17,95; 60,5 |
| *D. majalis ssp. traunsteineri* | Austria | 12,3833333333333; 47,45 |
| *D. majalis ssp. traunsteineri* | Austria | 15,3333333333333; 47,75 |
| *D. majalis ssp. traunsteineri* | Finland | 28,05; 64,3166666666667 |
| *D. majalis ssp. traunsteineri* | Finland | 24,85; 67,5333333333333 |
| *D. majalis ssp. traunsteineri* | Finland | 29,95; 64,75 |
| *D. majalis ssp. traunsteineri* | Finland | 26,8833333333333; 67,6 |
| *D. majalis ssp. traunsteineri* | Finland | 25,3833333333333; 67,6166666666667 |
| *D. majalis ssp. traunsteineri* | Finland | 25,4166666666667; 65,2166666666667 |
| *D. majalis ssp. traunsteineri* | Finland | 25,0333333333333; 66,0833333333333 |
| *D. majalis ssp. traunsteineri* | Lithuania | 21,4666666666667; 55,9166666666667 |
| *D. majalis ssp. traunsteineri* | Lithuania | 21,0666666666667; 55,8833333333333 |
| *D. majalis ssp. traunsteineri* | Norway | 10,3; 59,8166666666667 |
| *D. majalis ssp. traunsteineri* | Norway | 10,1; 59,7166666666667 |
| *D. majalis ssp. traunsteineri* | Norway | 10,85; 59,7666666666667 |
| *D. majalis ssp. traunsteineri* | Norway | 14,4833333333333; 67,3333333333333 |
| *D. majalis ssp. traunsteineri* | Norway | 9,5; 59,2666666666667 |
| *D. majalis ssp. traunsteineri* | Norway | 9,81666666666667; 59,3 |
| *D. majalis ssp. traunsteineri* | Norway | 9,5; 59,2666666666667 |
| *D. majalis ssp. traunsteineri* | Norway | 10,1666666666667; 59,5 |
| *D. majalis ssp. traunsteineri* | Norway | 10,1666666666667; 59,5 |
| *D. majalis ssp. traunsteineri* | Norway | 14,0666666666667; 67,0333333333333 |
| *D. majalis ssp. traunsteineri* | Norway | 14,0166666666667; 67,0333333333333 |
| *D. majalis ssp. traunsteineri* | Russia | 33,8; 61,7833333333333 |
| *D. majalis ssp. traunsteineri* | Sweden | 16,6; 63,2666666666667 |
| *D. majalis ssp. traunsteineri* | Sweden | 17,1833333333333; 63,5833333333333 |
| *D. majalis ssp. traunsteineri* | Sweden | 16,4166666666667; 63,5166666666667 |
| *D. majalis ssp. traunsteineri* | Sweden | 15,9666666666667; 63,6166666666667 |
| *D. majalis ssp. traunsteineri* | Sweden | 17,1333333333333; 61,0333333333333 |
| *D. majalis ssp. traunsteineri* | Sweden | 18,65; 57,8166666666667 |
| *D. majalis ssp. traunsteineri* | Sweden | 18,7; 57,9166666666667 |
| *D. majalis ssp. traunsteineri* | Sweden | 18,8333333333333; 57,8166666666667 |
| *D. majalis ssp. traunsteineri* | Sweden | 18,8833333333333; 57,8166666666667 |
| *D. majalis ssp. traunsteineri* | Sweden | 18,3166666666667; 57,3333333333333 |
| *D. majalis ssp. traunsteineri* | Sweden | 14,9; 61,6166666666667 |
| *D. majalis ssp. traunsteineri* | Sweden | 16,8666666666667; 61,2666666666667 |
| *D. majalis ssp. traunsteineri* | Sweden | 16,7333333333333; 61,5333333333333 |
| *D. majalis ssp. traunsteineri* | Sweden | 16,8333333333333; 61,4666666666667 |
| *D. majalis ssp. traunsteineri* | Sweden | 15,0166666666667; 63,3166666666667 |
| *D. majalis ssp. traunsteineri* | Sweden | 15,7; 62,5666666666667 |
| *D. majalis ssp. traunsteineri* | Sweden | 17,45; 62,25 |
| *D. majalis ssp. traunsteineri* | Sweden | 14,9333333333333; 58,45 |
| *D. majalis ssp. traunsteineri* | Sweden | 15,5333333333333; 58,4166666666667 |
| *D. majalis ssp. traunsteineri* | Sweden | 23,2333333333333; 67,2833333333333 |
| *D. majalis ssp. traunsteineri* | Sweden | 20,45; 64,9333333333333 |
| *D. majalis ssp. traunsteineri* | Sweden | 14,7666666666667; 57,6333333333333 |
| *D. majalis ssp. traunsteineri* | Sweden | 17,1166666666667; 58,75 |
| *D. majalis ssp. traunsteineri* | Sweden | 18,3; 59,9833333333333 |
| *D. majalis ssp. traunsteineri* | Sweden | 18,3166666666667; 60,2833333333333 |
| *D. majalis ssp. traunsteineri* | Sweden | 17,6166666666667; 60,6166666666667 |
| *D. majalis ssp. traunsteineri* | Sweden | 19,0333333333333; 59,7 |
| *D. majalis ssp. traunsteineri* | Sweden | 18,6666666666667; 59,8833333333333 |
| *D. majalis ssp. traunsteineri* | Sweden | 18,3166666666667; 59,7333333333333 |
| *D. majalis ssp. traunsteineri* | Sweden | 14,6833333333333; 58,3166666666667 |
| *D. majalis ssp. traunsteineri* | Sweden | 13,7666666666667; 58,3166666666667 |
| *D. majalis ssp. traunsteineri* | Sweden | 14,7666666666667; 59,4 |
| *D. majalis ssp. traunsteineri* | Sweden | 14,7833333333333; 59,4333333333333 |
| *D. majalis ssp. traunsteineri* | Sweden | 17,1833333333333; 63,5833333333333 |
| *D. majalis ssp. traunsteineri* | Sweden | 18,6666666666667; 57,6833333333333 |
| *D. majalis ssp. traunsteineri* | Sweden | 18,35; 57,5833333333333 |
| *D. majalis ssp. traunsteineri* | England | 2,21666666666667; 53,9833333333333 |
| *D. majalis ssp. traunsteineri* | Scotland | 5,55; 57,7666666666667 |
