# Supplementary material for: Glacial Refugia and Future Habitat Coverage of Selected Dactylorhiza Representatives (Orchidaceae)
Source: PLoS One. 2015 Nov 23;10(11):e0143478. doi: 10.1371/journal.pone.0143478 (PMC4657909; doi:10.1371/journal.pone.0143478)
Supplement: S2 Table — Standard deviation values are given in parenthesis. (DOC) [file pone.0143478.s003.doc]

**S2 Table. The average training AUC for the replicate runs measured for Last Glacial Maximum (LGM), future climate change scenarios (A1b, A2a, B2a) and present time models. Standard deviation values are given in parenthesis.**

| **Taxon** | **LGM**  **(PMIP2^a^)** | **LGM (CMIP5^b^)** | **A1b** | **A2a** | **B2a** | **Present time** |
| --- | --- | --- | --- | --- | --- | --- |
| *D. incarnata* var. *cruenta* | 0.997 (0.001) | 0.997 (0.001) | 0.997 (0.001) | 0.998  (0.001) | 0.997 (0.001) | 0.997 (0.001) |
| *D. incarnata* var. *incarnata* | 0.991 (0.001) | 0.992 (0.001) | 0.992 (0.001) | 0.993 (0.001) | 0.992 (0.001) | 0.992 (0.001) |
| *D. incarnata* var. *ochroleuca* | 0.998 (0.001) | 0.998 (0.001) | 0.998 (0.001) | 0.998 (0.001) | 0.998 (0.001) | 0.998 (0.001) |
| *D. maculata* ssp. *fuchsii* | 0.989 (0.001) | 0.990 (0.001) | 0.990 (0.001) | 0.991 (0.001) | 0.989 (0.001) | 0.989 (0.001) |
| *D. maculata* ssp. *maculata* | 0.987 (0.001) | 0.988 (0.001) | 0.989 (0.000) | 0.989 (0.001) | 0.988 (0.001) | 0.987 (0.001) |
| *D. majalis* ssp. *lapponica* | 0.996 (0.001) | 0.995 (0.001) | 0.996 (0.001) | 0.996 (0.001) | 0.996 (0.001) | 0.996 (0.001) |
| *D. majalis* ssp. *majalis* | 0.996 (0.001) | 0.997 (0.001) | 0.997 (0.000) | 0.997 (0.000) | 0.997 (0.000) | 0.996 (0.000) |
| *D. majalis* ssp. *traunsteineri* | 0.994 (0.001) | 0.995 (0.001) | 0.994 (0.001) | 0.994 (0.001) | 0.995 (0.001) | 0.994 (0.001) |

^a^ paleo-environmental data sets developed by Paleoclimate Modelling Intercomparison Project Phase

^b^ paleo-environmental data sets developed by Coupled Model Intercomparison Project Phase
