# Supplementary figures and images for: Glacial Refugia and Future Habitat Coverage of Selected Dactylorhiza Representatives (Orchidaceae)
Source: PLoS One. 2015 Nov 23;10(11):e0143478. doi: 10.1371/journal.pone.0143478 (PMC4657909; doi:10.1371/journal.pone.0143478)

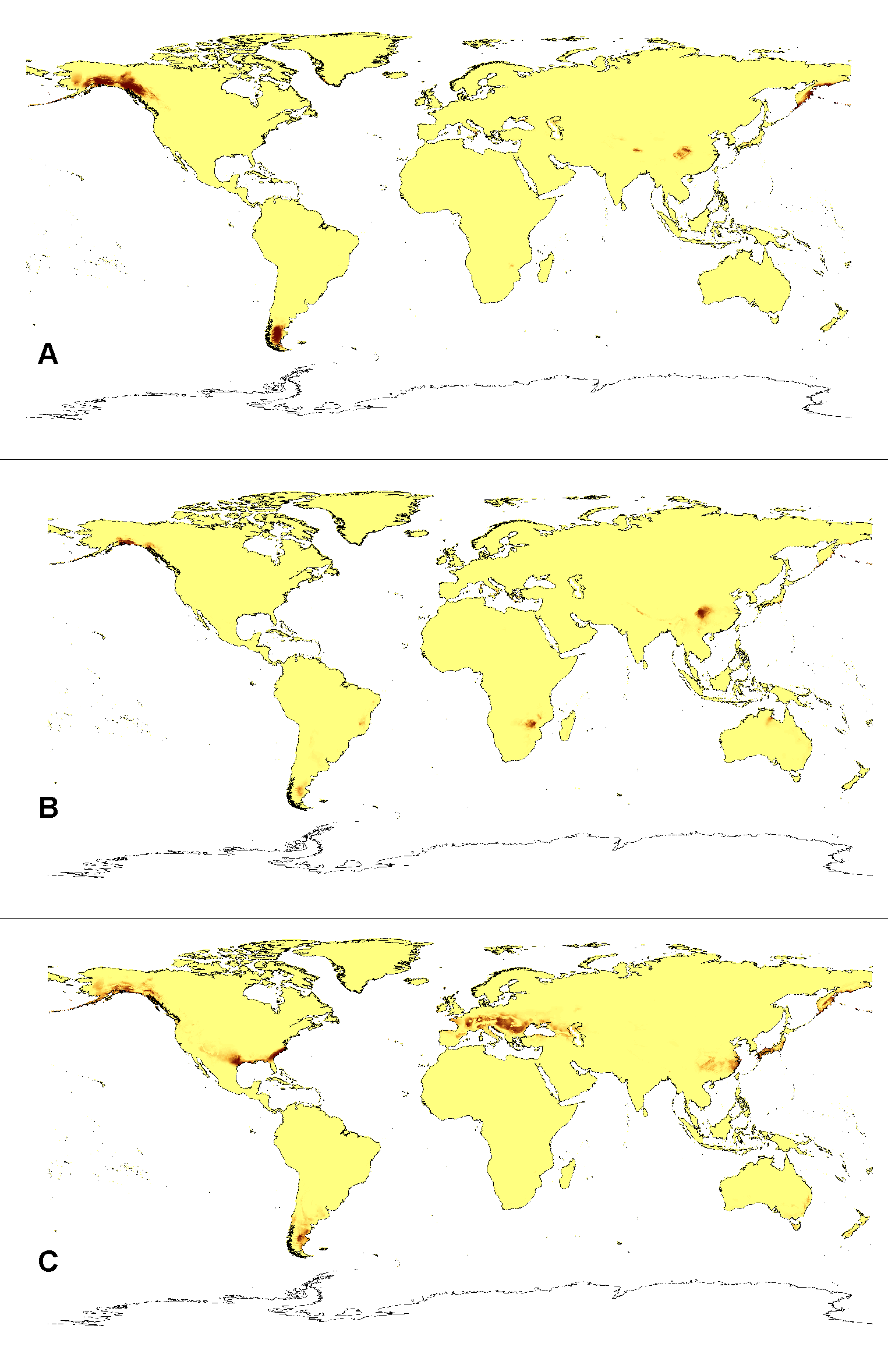

Supplement: S1 Fig — (TIF) [file pone.0143478.s004.tif]

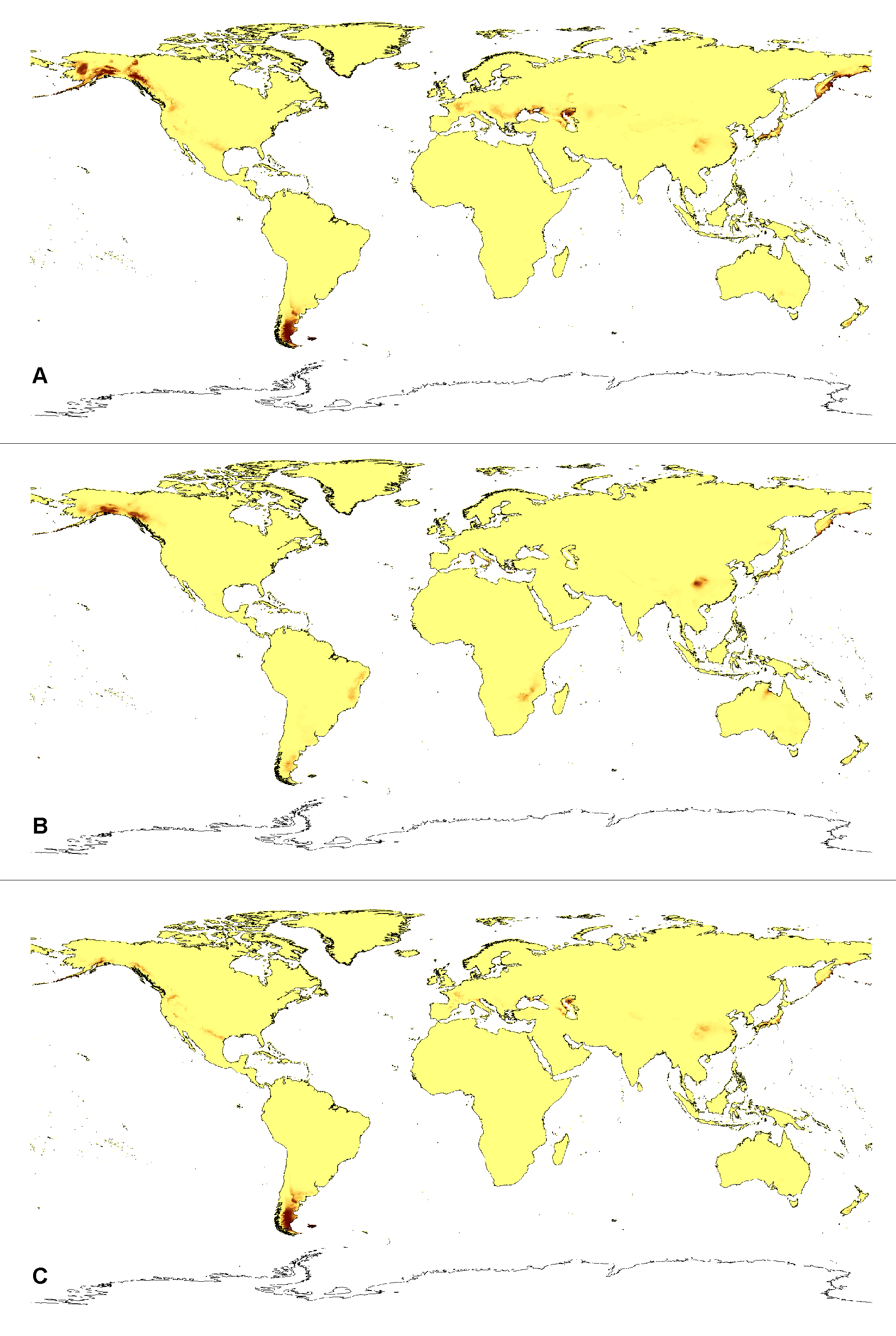

Supplement: S2 Fig — (TIF) [file pone.0143478.s005.tif]

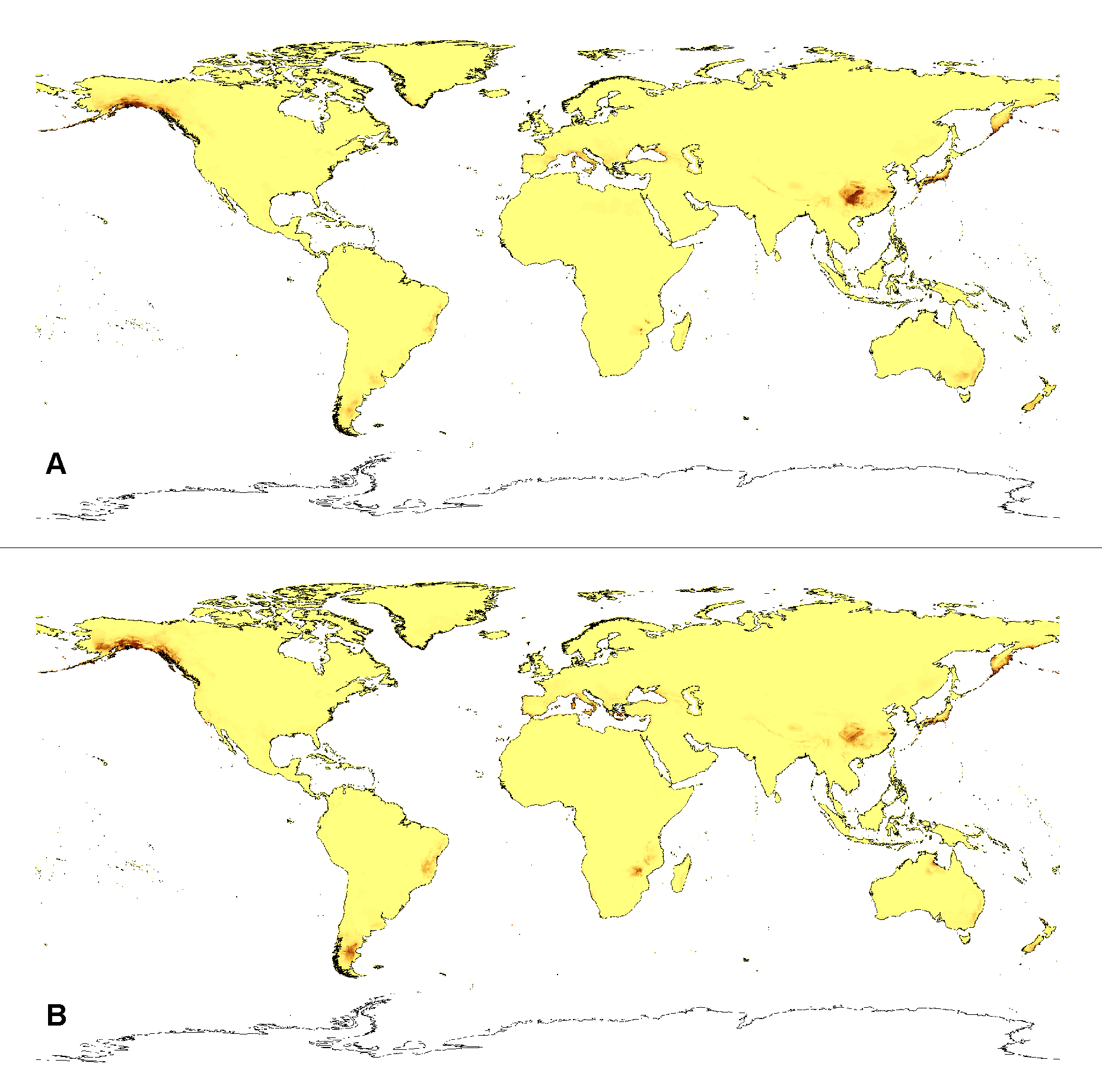

Supplement: S3 Fig — (TIF) [file pone.0143478.s006.tif]

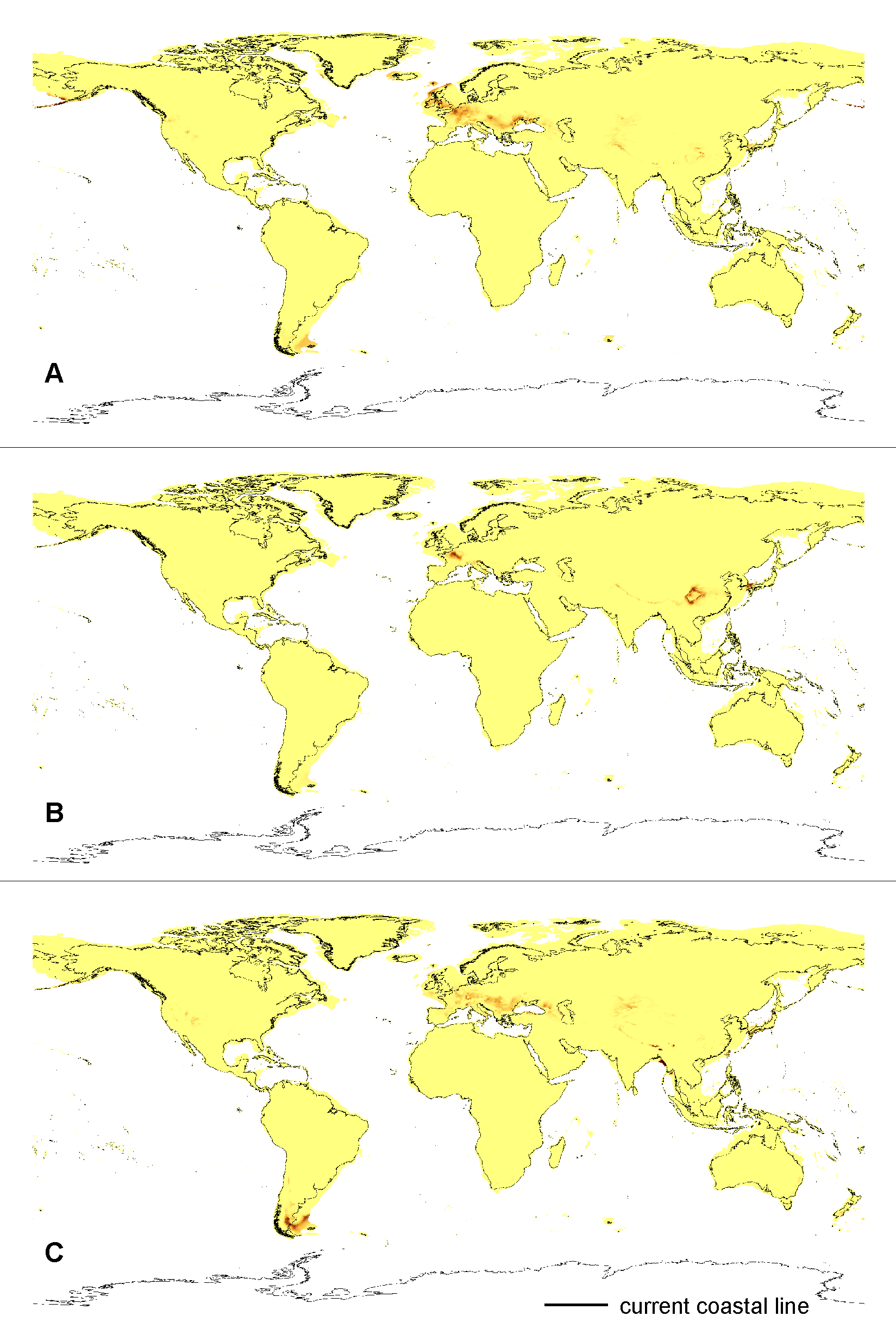

Supplement: S4 Fig — The current coastal line is indicated with a continuous line. (TIF) [file pone.0143478.s007.tif]

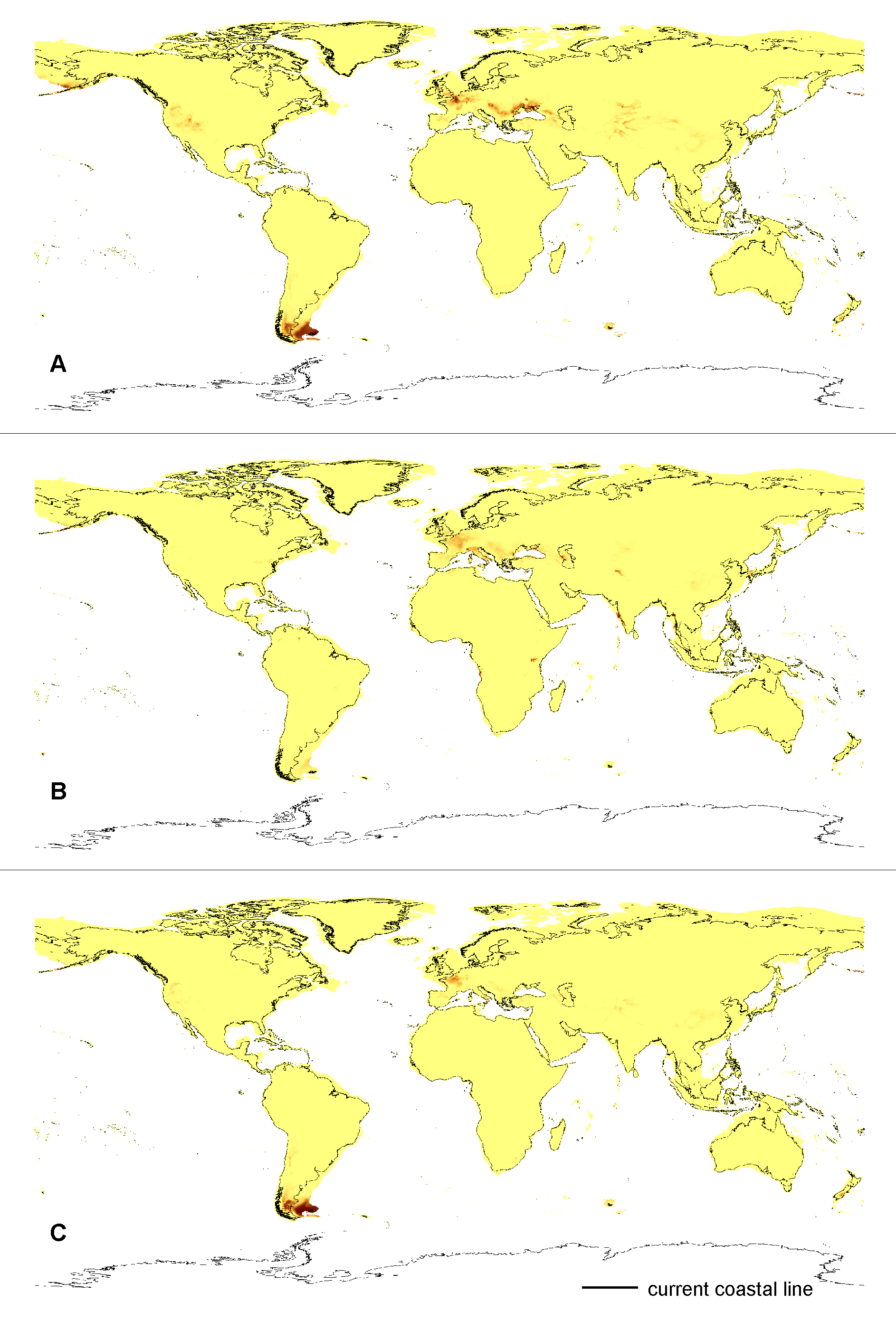

Supplement: S5 Fig — The current coastal line is indicated with a continuous line. (TIF) [file pone.0143478.s008.tif]

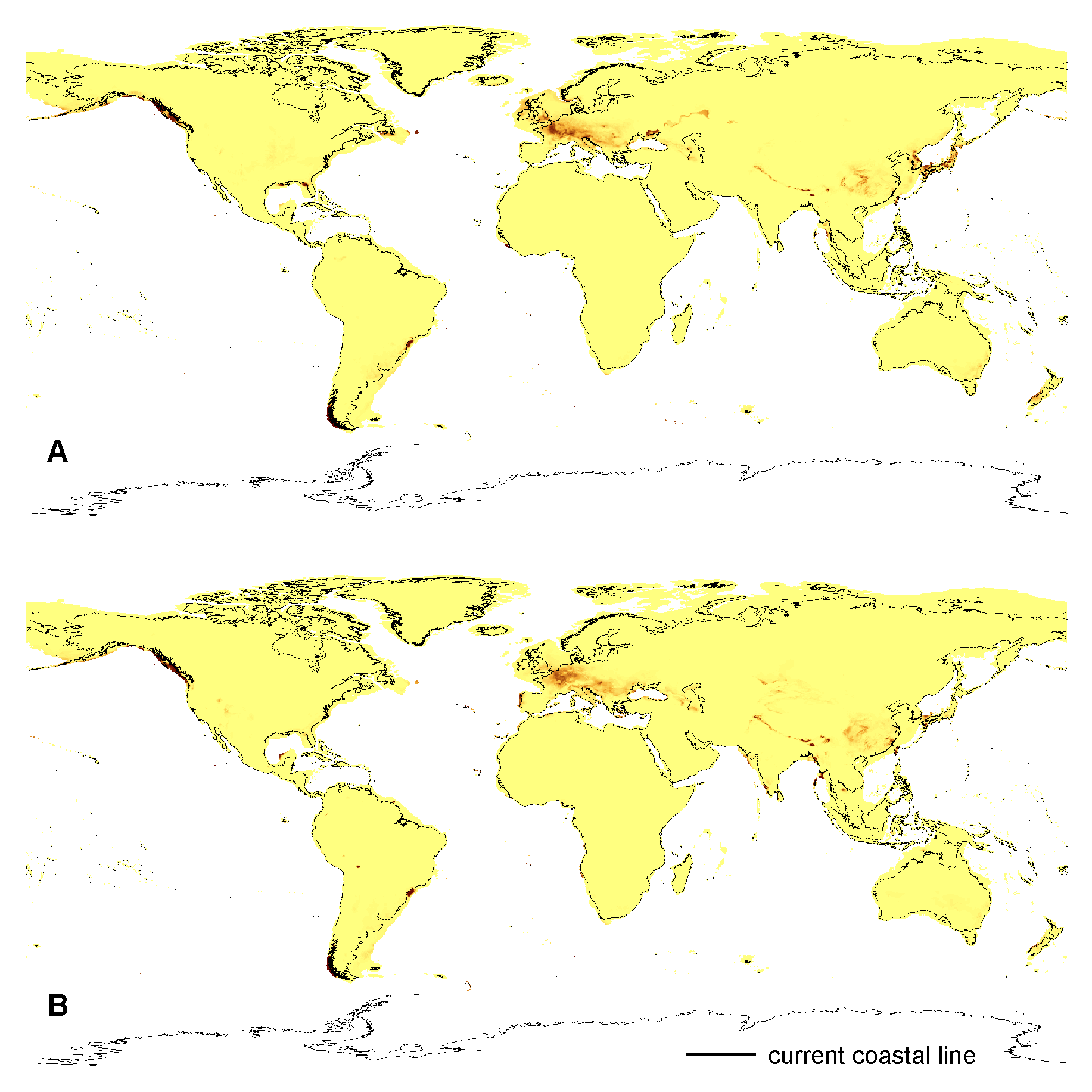

Supplement: S6 Fig — The current coastal line is indicated with a continuous line. (TIF) [file pone.0143478.s009.tif]

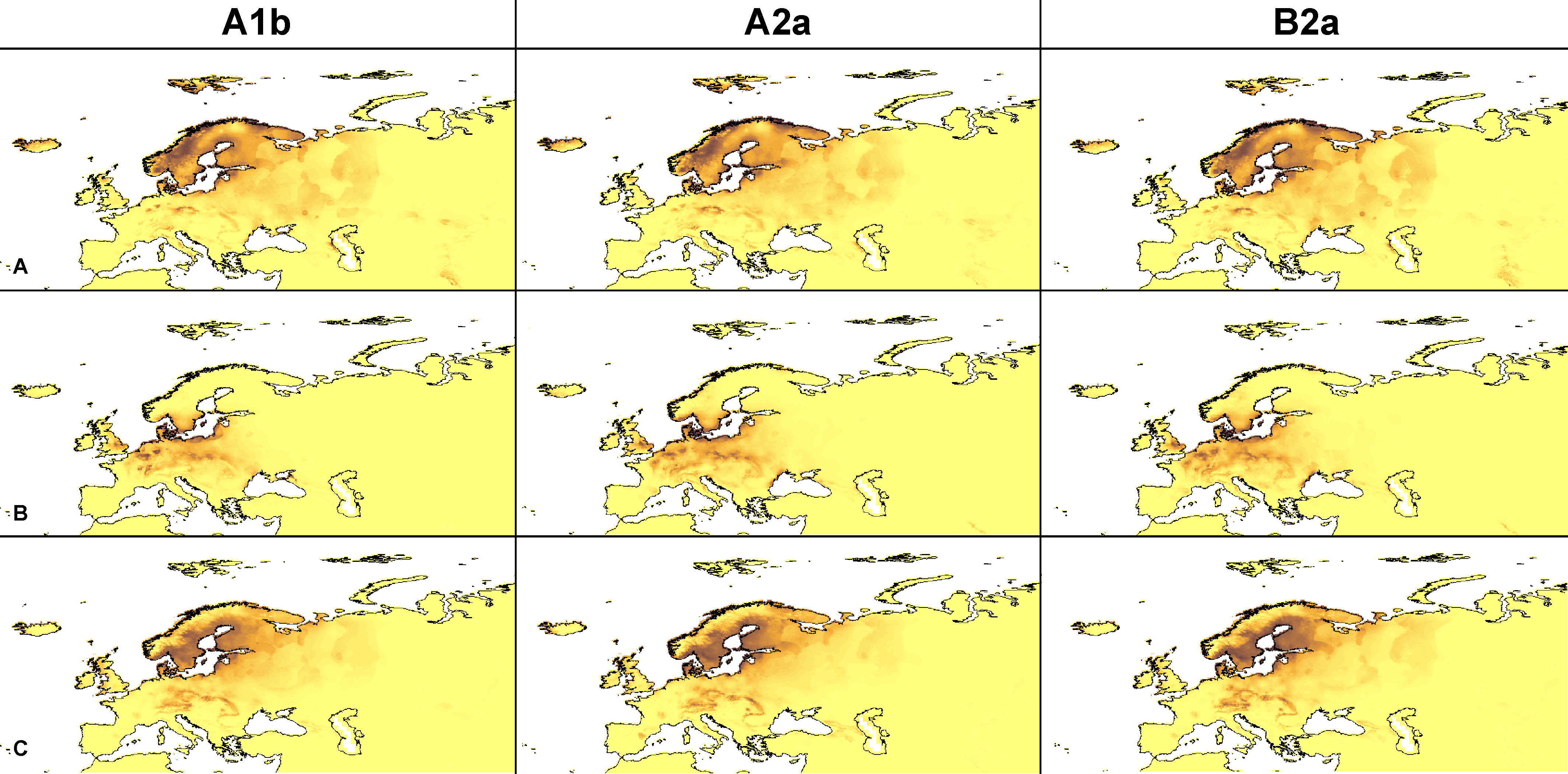

Supplement: S7 Fig — (TIF) [file pone.0143478.s010.tif]

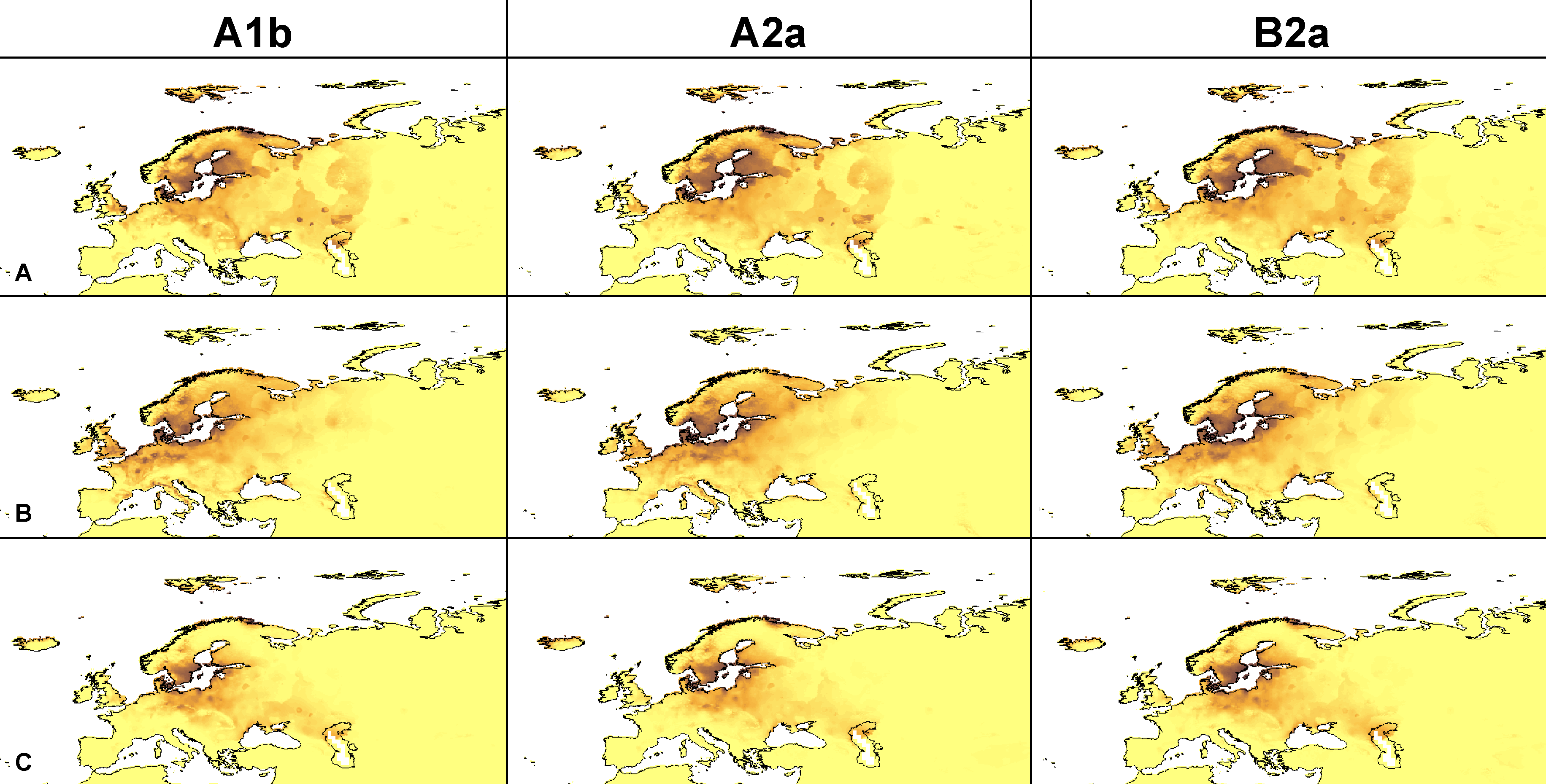

Supplement: S8 Fig — (TIF) [file pone.0143478.s011.tif]

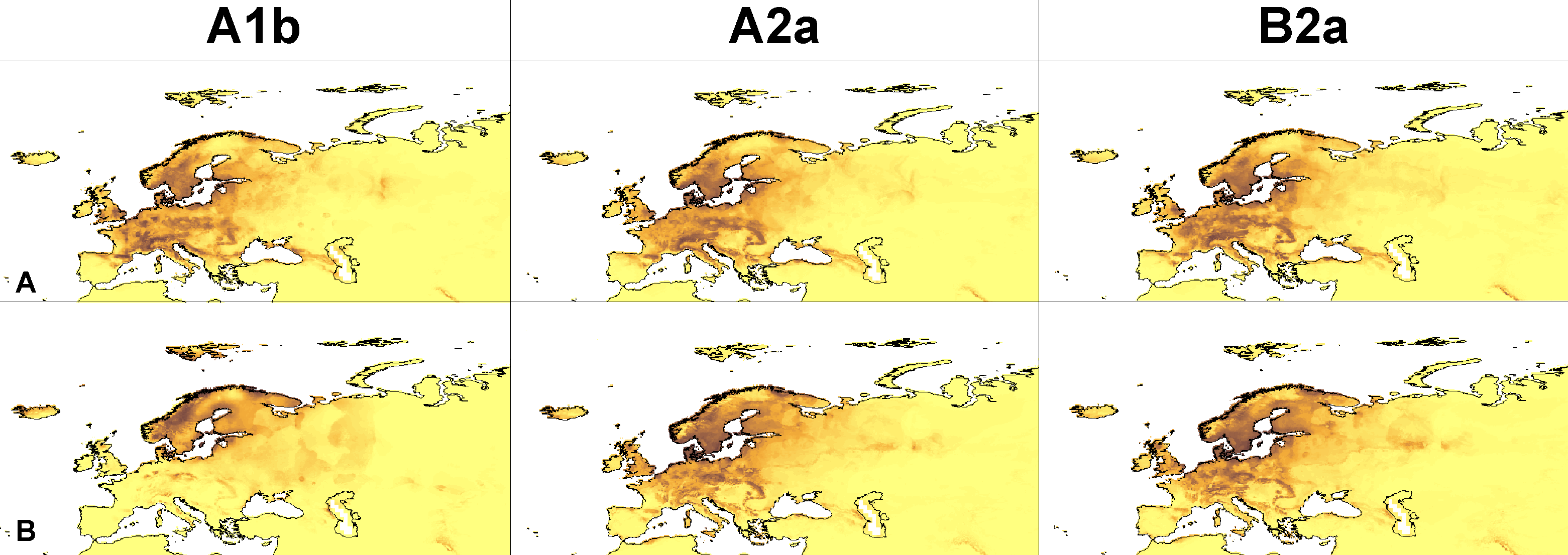

Supplement: S9 Fig — (TIF) [file pone.0143478.s012.tif]
